# *Arabidopsis* acclimation to daily environmental fluctuations converts a defense response regulator into a susceptibility factor toward *Sclerotinia*

**DOI:** 10.1111/nph.71053

**Published:** 2026-03-07

**Authors:** Marie Didelon, Justine Sucher, Florent Delplace, Pedro Carvalho‐Silva, Matilda Zaffuto, Adelin Barbacci, Sylvain Raffaele

**Affiliations:** ^1^ Laboratoire des Interactions Plantes‐Microbes‐Environnement (LIPME) Université de Toulouse, INRAE, CNRS F‐31326 Castanet‐Tolosan France

**Keywords:** acclimation, *Arabidopsis thaliana*, fungal pathogen, plant immunity, priming, *Sclerotinia sclerotiorum*, transcription factor

## Abstract

- Acclimation enables plants to adjust to immediate environmental fluctuations and is therefore key to the resilience of plant disease resistance in a time of climate change. Here, we report on the acclimation of *Arabidopsis thaliana* quantitative immune responses against the fungal pathogen *Sclerotinia sclerotiorum* to daily environmental fluctuations.
- We analyzed disease resistance phenotypes and global gene expression in plants grown in three acclimation regimes, revealing the rewiring of regulatory networks during this process. We identified pathogen‐induced genes weakly sensitive to acclimation as promising bases for acclimation‐proof immunity.
- Fluctuations in Mediterranean‐like acclimation resulted in an increased disease susceptibility and the misregulation of many pathogen‐responsive genes. We identified *A. thaliana* mutants in novel immune components contributing positively to quantitative disease resistance following temperate but not Mediterranean acclimation. Quantitative disease resistance was maintained under Mediterranean acclimation in *NAC42‐like* mutants and associated with a switch in the repertoire of pathogen‐responsive targets of this transcription factor.
- Our work reveals the role of immune gene networks' plasticity in acclimation and suggests new strategies to maintain plant immune function in a warming climate.

## Introduction

The ability of species to cope with rising temperatures is a crucial factor influencing range shifts and local extinctions, as their distribution and range boundaries closely align with temperature gradients. Evidence shows that plant species adapt to local environmental conditions through genetic variation (Fournier‐Level *et al*., 2011; Katz *et al*., 2021; Clauw *et al*., 2022), but they also exhibit phenotypic plasticity, allowing individual plants to adjust rapidly their physiology to environmental variations (Valladares *et al*., 2014; Brancalion *et al*., 2018). The short‐term, reversible process that allows plants to cope with immediate environmental fluctuations is often referred to as acclimation (Kleine *et al*., 2021). Plant acclimation helps maintain the balance of natural systems, supporting biodiversity and the services that ecosystems provide, such as carbon sequestration and water regulation. With climate change modifying the distribution area of plants (Sloat *et al*., 2020) and causing more frequent and severe weather events (Newman & Noy, 2023), knowledge of how plants acclimate can inform strategies to manage ecosystems and agriculture. In this context, crops that can acclimate effectively are more likely to maintain high yields despite stressors. A better understanding of the genetic underpinnings of plant acclimation is therefore crucial for predicting the impact of climate change on ecosystems and for improving crop resilience.

Acclimation differs from adaptation in involving changes to the expression of the genome instead of heritable changes to genome sequences (Kleine *et al*., 2021). Epigenetic and transcriptional regulation mechanisms mediating somatic stress memory are important players in plant acclimation (Charng *et al*., 2023; Zuo *et al*., 2023; Hadj‐Amor *et al*., 2024; Zarattini & Fagard, 2025). Cold acclimation, by which decreasing temperatures enhance freezing tolerance in plants, involves alterations in membrane composition, the production of cryoprotective polypeptides and solutes and the activation of cold‐responsive (COR) genes regulated by C‐repeat binding transcription factors (CBFs/DREB1) (Liu *et al*., 2019a). The accumulation of heat shock proteins regulated by heat shock transcription factors and histone 3 K4 methylation plays a key role in heat acclimation (Nishad & Nandi, 2021; Kappel *et al*., 2023). Besides transcription factors and epigenetic marks, the hormone abscisic acid (ABA) is a central mediator of the accumulation of LEA‐like protective proteins, stomatal closure and downregulation of photosynthesis under drought acclimation (Sadhukhan *et al*., 2022). Despite recent efforts, the interplay between regulatory mechanisms, molecular and phenotypic responses to plant acclimation is elusive.

With changes to the climate, not only does the distribution range of plants change, but also that of their enemies. Suitable conditions for plant disease outbreaks are expected to shift in time and space, leading to a global poleward movement of plant‐pathogen geographic niches (Bebber *et al*., 2013) and an increased risk of infection by pathogenic fungi and oomycetes (Chaloner *et al*., 2021). Fungi, especially generalists with a broad range of plant hosts, are the most widespread and most rapidly spreading pathogens, so that if current rates persist, several major food‐producing countries would have fully saturated pathogen distributions by 2050 (Bebber *et al*., 2014). A paradigmatic example of such a broad host range pathogen is the white and stem mold necrotrophic fungus *Sclerotinia sclerotiorum*, which infects hundreds of plant species and causes significant losses to vegetable and oil crops world‐wide (Peltier *et al*., 2012; Navaud *et al*., 2018; Cohen, 2023). Although climate change may alter the overlap between crop cultivation area and *S. sclerotiorum* distribution range (Mehrabi *et al*., 2019), pathogen strains adapted to warm temperatures have been reported (Uloth *et al*., 2015) and extreme temperatures may promote fungal development (Lane *et al*., 2019; Shahoveisi *et al*., 2022), raising concern about *Sclerotinia* disease incidence in the future (Singh *et al*., 2023).

Plants respond to *S. sclerotiorum* through quantitative disease resistance (QDR), a complex immune response resulting from the combined action of multiple genes of weak to moderate individual phenotypic effect (Roux *et al*., 2014; Sucher *et al*., 2020; Delplace *et al*., 2025). The diverse molecular components contributing to QDR against *S. sclerotiorum* include immune receptors, reactive oxygen species, phytohormones such as ABA, jasmonic acid and ethylene, transcription factors and phytoalexins (Perchepied *et al*., 2010; Mbengue *et al*., 2016; Derbyshire & Raffaele, 2023). Several of these components not only contribute to plant immunity but are also involved in diverse biological processes (BP), such as plant development and response to the abiotic environment (Corwin *et al*., 2016; Badet *et al*., 2019; Léger *et al*., 2022). The multigenic nature of QDR suggests that the expression of many genes contributing to QDR could be modulated by environmental conditions (Hadj‐Amor *et al*., 2024) and that climate change may alter plant QDR response to *S. sclerotiorum* at the phenotypic and molecular level. Temperature increase, notably, is known to frequently impair plant immune responses, including QDR (Zhu *et al*., 2010; Aoun *et al*., 2017; Desaint *et al*., 2021; Zarattini & Fagard, 2025).

Analyses of plant immune responses under abiotic constraints generally focus on pathogen inoculation under prolonged and stable abiotic conditions. In *A. thaliana*, immunity against the bacterial pathogen *Pseudomonas syringae* pv *tomato* at elevated temperature can be restored by the constitutive expression of *CBP60g*, a major transcriptional regulator of plant immunity genes and salicylic acid (SA) defense hormone production, downregulated by temperature (Kim *et al*., 2022). This finding indicates that engineering plant transcriptional circuits can mitigate the negative effect of climate change on some plant immune responses. Whether this strategy would restore QDR against necrotrophic pathogens such as *S. sclerotiorum* remains to be determined. Another promising target is the disordered protein TWA1, a temperature sensor proposed to orchestrate acclimation by integrating temperature with ABA and JA signaling (Bohn *et al*., 2024), which play important roles in plant defense against necrotrophs. When applied sequentially, prior abiotic signals may alter the transcriptional and metabolic response to a subsequent pathogen inoculation (Coolen *et al*., 2016; Garcia‐Molina *et al*., 2020; Garcia‐Molina & Pastor, 2024). In addition to mean temperature increase, climate change drives an expansion of diurnal temperature range (Zhong *et al*., 2023). Daily fluctuations of the environment may alter plant metabolism, growth and flowering (Burghardt *et al*., 2016; Matsubara, 2018; Deng *et al*., 2021) as well as gene regulation and invasive growth of filamentous pathogens (Wang *et al*., 2011; Jallet *et al*., 2020; Bernard *et al*., 2022). Climate change not only modifies local average temperatures but also alters day–night temperature fluctuations and the range of plant species distribution, creating a need for understanding how plants adjust to environmental fluctuation representative of major climate zones. Yet, how acclimation to daily environmental fluctuations modifies plant quantitative immunity to *S. sclerotiorum* is largely unexplored. In particular, which genetic responses to fungal inoculation remain functional under diverse environmental fluctuation scenarios remains to be determined.

To fill this gap, we analyzed *A. thaliana* immune responses upon *S. sclerotiorum* inoculation following three acclimation regimes simulating conditions likely to occur through the distribution area of these two species. We varied day temperature (11°C, 20°C and 23°C, respectively), night temperature (1°C, 9°C and 7°C) and daylength (14, 13 and 13 h, respectively) for acclimation we designated as continental, temperate and Mediterranean respectively. Mediterranean acclimation caused an increase in disease susceptibility in the three natural accessions we tested, while temperate and continental acclimation resulted in similar disease susceptibility levels. Using global gene expression analyses, we show that acclimation alters the expression of nearly half of pathogen‐responsive genes, many of which are downregulated by inoculation and associated with disease susceptibility. The phenotypic analysis of *A. thaliana* mutants identified novel components of QDR following temperate acclimation. Several of these mutants were, however, more resistant than wild‐type following Mediterranean acclimation. In particular, contrary to wild‐type, two mutant lines in the NAC42‐like transcription factor showed no loss of QDR upon Mediterranean acclimation. These phenotypes are associated with a switch in the repertoire of NAC42‐like targets differentially regulated by inoculation according to acclimation. These findings reveal the rewiring of immune gene regulatory networks by acclimation and open new perspectives to safeguard the functioning of the plant immune system in a warming climate.

## Materials and Methods

### Plant material and growth conditions

*Arabidopsis thaliana* (L.) Heynh. natural accessions and mutant lines were obtained from the Nottingham Arabidopsis Stock Center. We selected Col‐0 (CS76778, 6909), Rld‐2 (CS78349, 7457) and Shahdara (Sha, CS78397, 6962) as three natural accessions of *A. thaliana* originating from areas with contrasted climate conditions. The genotype of accessions was verified by sequencing PCR products obtained using primers Fw‐1 and Rv‐1, Fw‐2 and Rv‐2 and Fw‐3 and Rv‐3 (Supporting Information Table S1). Plants were grown in jiffy pots for 35 or 70 d in Percival E41‐L3 and E41‐L2PLT growth cabinets equipped with an ultrasonic humidifier, far‐red LED clusters, closed‐loop light dimming, Intellus and WeatherEZE controllers. We set day and night temperatures for each climate according to ERA5T models based on 30‐yr average of hourly weather simulations for daily maximum and minimum temperatures for the month of April at GPS coordinates 56.25°N, 34.19°E (Continental climate, origin of Rld‐2 accession); 38.35°N, 68.48°E (Mediterranean climate, origin of Sha accession) and 38.30°N, 92.30°O (Temperate climate) according to https://www.meteoblue.com consulted on April 2017 (Fig. S1). Day temperatures were 11°C, 20°C and 23°C and night temperatures were 1°C, 9°C and 7°C for continental, temperate and Mediterranean climates, respectively. Plants were grown in long day under 190 μmol m^−2^ s^−1^ light, with photoperiod variation between climates to represent photoperiod variability during April in the northern hemisphere; water was kept non‐limiting for the whole experiment at 80% relative humidity and soil moisture was adjusted to 65 ± 5% relative humidity. These growth conditions were classified into Continental, Mediterranean and Temperate according to Köppen‐Geiger classification (Cui *et al*., 2021) of climate at the corresponding GPS coordinates.

### Fungal strains and disease resistance phenotyping

For disease phenotyping, inoculations were performed on detached leaves at a constant 23°C under constant 40 μmol m^−2^ s^−1^ light and high humidity following the procedure described in Barbacci *et al*. (2020). All inoculations were performed at the middle of the daytime period, corresponding to 6.5, 6.5 and 7 h after the switch to day conditions for temperate, Mediterranean and continental acclimation, respectively. 0.5‐cm‐wide plugs of PDA agar medium containing *Sclerotinia sclerotiorum* (Libert) de Bary strain 1980 UF‐1, grown for 72 h at 20°C on 14 cm Petri dishes, were placed on the adaxial surface of detached leaves. Records were made using high‐definition (HD) cameras ‘3MP M12 HD 2.8–12 mm 1/2.5 IR 1 : 1.4 CCTV Lens’ every 10 min. For each genotype, a minimum of 28 leaves were imaged from a minimum of two independent acclimation and inoculation experiments. Kinetics of *S. sclerotiorum* disease lesions were analyzed using Infest script v.1.0 (https://github.com/A02l01/INFEST). The slope of the disease lesion over time was ‐log transformed into a ‘Quantitative Resistance’ metric expressed in minutes, representing the characteristic time for lesion increase (high values correspond to slower colonization of the fungus). Statistical analyses of disease phenotypes were conducted using the Tukey test or the Student's *t* test, followed by Benjamini–Hochberg correction for multiple testing in R 4.2.1.

### RNA collection and sequencing

For RNA collection, inoculations were performed as described above on whole plants placed at 23°C constant and high humidity. Inoculated and non‐inoculated samples were harvested at the same time. Total RNA was extracted from a 3 mm‐wide ring of leaf tissue at the edge of the *c*. 1.5 cm wide disease lesions collected at 30 h post inoculation (hpi) for temperate and Mediterranean acclimation and 48 hpi for continental acclimation. The samples were harvested with a scalpel on a cool glass slide and immediately frozen in liquid nitrogen. Samples were ground with metal beads (2.5 mm) in a Retschmill apparatus (24 Hz for 2 × 1min). RNA was extracted using the RNAplus kit (Macherey Nagel, Dueren, Germany) following the manufacturer's instructions. A Turbo DNAse treatment (Ambion, Carlsbad, CA, USA) was applied to remove genomic DNA. The quality and concentrations of RNA preparations were assessed with an Agilent Bioanalyzer using the Agilent RNA 6000 Nano kit. All samples had an RNA integrity index (RIN) between 5.0 and 8.5, except for four samples with a RIN between 4.3 and 4.9. For the analysis of gene expression in natural accessions, library synthesis and sequencing were outsourced to Fasteris SA (Plan‐les‐Ouates, Switzerland). Libraries were sequenced as 125 bp paired‐end reads on an Illumina HiSeq 2500 instrument in High Output v.4 mode with 2 × 125 + 8 cycles on seven lanes of HiSeq Flow Cells v.4 with the HiSeq SBS Kit v.4. Basecalling was performed with the HiSeq Control Software 2.2.58, Rta 1.18.64.0 and Casava‐1.8.2. Reads QC was performed using spiked‐PhiX in‐lane controls, yielding a Q30 error rate < 0.4% for all lanes. We obtained 21.7–40.2 million reads per sample. Paired‐end reads were trimmed and mapped to the TAIR10.0 reference genome using the RNA‐seq analysis tool of the CLC Genomics Workbench 11.0.1 software (Qiagen). The following mapping parameters were used: mismatch cost 2, insertion cost 3, deletion cost 3, length fraction 0.8, similarity fraction 0.8, both strands mapping and 10 hits maximum per read, with expression value given as total read count per gene.

### Differential expression and expression variance analyses

Differential gene expression analysis was performed with the DESeq2 Bioconductor package v.1.8.2 (Love *et al*., 2014) in R 3.4.0 in a pairwise manner using expression in uninfected plants as a reference with ~replicates + inoculation as the design formula. Genes with baseMean 0 in all differential comparisons and non‐nuclear genes were discarded from further analyses. Genes with |log_2_ fold change (LFC)| ≥ 2 and Bonferroni‐adjusted *P*‐val < 0.001 in DESeq2 Wald test were considered significant for differential expression. For ANOVA, read counts were mean‐normalized to homogenize the total number of mapped reads per sample. The ANOVA was performed on each gene using the dplyr package in R with ReadCount ~ Genotype * Infection * Climate as the model formula. *P*‐values associated with each factor were corrected for multiple testing using the Benjamini‐Hochberg procedure.

### Gene network reconstruction and analyses

For gene network reconstruction, we focused on the 7279 genes whose expression was pathogen inoculation‐dependent both in the differential analysis (|LFC| ≥ 2 and Bonferroni‐adjusted *P*‐val < 1E‐03) and in the ANOVA analysis (Benjamini‐Hochberg corrected *P*‐val < 1E‐05). Pairwise Spearman rank correlation was calculated for the expression of these genes using the rcorr function from the R package hmisc, using the raw counts per gene from all 54 RNA‐seq samples. The top 25 correlated genes (Spearman ρ > 0.85) were extracted for each gene. The top 25 expression correlations were used as edges for network reconstruction with weight ρ. Reconstruction of the hierarchical gene cluster network was performed using the Community Detection 1.12.0 plugin in Cytoscape 3.10.0, using the HiDeF algorithm with maximum resolution 45.0, consensus threshold 65, persistent threshold 6 and the Louvain algorithm. Correlation with susceptibility was the Spearman rank correlation coefficient between normalized read counts (averaged over three replicates) for each gene and the slope of disease lesion growth. Average LFC was the mean LFC over all nine genotype‐acclimation modalities tested. Values for gene clusters are the mean of values for all genes in a cluster. Gene ontology enrichment was analyzed with the BinGO plugin in Cytoscape 3.10.0 using a hypergeometric test with Benjamini and Hochberg false discovery rate correction, at significance level 0.05 with *A. thaliana* whole annotation as a reference set.

### Characterization of *A. thaliana* mutant lines

T‐DNA insertion lines in AT1G76600 (SALK_052389), AT1G34190 (SALK_044777), AT1G07135 (SALK_133656), AT2G43790 (mpk6‐1), AT5G24600 (SALK_201248C), AT1G12290 (SALK_125493), AT5G60600 (SALK_059118), AT5G23160 (SALK_041095C), AT5G64990 (SALK_088173), AT5G06230 (SALK_008492C), AT3G12910 (SALK_016619C and SALK_078841) and AT5G37840 (SALK_002404) in the Col‐0 background were obtained from the Nottingham Arabidopsis Stock Centre. To identify homozygous insertion lines, the lines were genotyped by PCR and, if needed, self‐crossed and the progeny genotyped by PCR. The disease resistance phenotype of mutant lines was analyzed as previously described in a total of 23 independent inoculation experiments each including the Col‐0 reference, with a minimum of two independent experiments for each mutant line. Primers used in all experiments are shown in Table S1. RNA for qRT‐PCR analysis was extracted from plants 24 h post inoculation with *S. sclerotiorum* as for RNA‐sequencing. For cDNA synthesis, 1 μg of RNA and 0.5 μl of Transcriptor reverse transcriptase (Roche) were used in a 20 μl reaction volume according to the manufacturer's protocol. The resulting cDNA diluted 1 : 10 served as the template for quantitative RT‐PCR. qRT‐PCR reactions were carried out with 5 pmol of specific oligonucleotides (Table S1), 2 μl of cDNA and 3.5 μl of SYBR GREEN I in a total volume of 7 μl. Amplification reactions were performed using a LightCycler 480 (Roche Diagnostics, Basel, Switzerland) with the following protocol: 9 min at 95°C, followed by 45 cycles of 5 s at 95°C, 10 s at 65°C and 20 s at 72°C. Relative gene expression was calculated as the ratio of target gene expression to the reference gene *AT2G28390* and expressed as the difference between target and reference crossing times (Δ*C*_t_).

### Identification of NAC42‐L predicted target genes

RNAs of inoculated *nac42‐L1* (SALK_016619C) and *nac42‐L2* (SALK_078841) plants were collected in triplicates 24 h post inoculation by *S. sclerotiorum* as described above. Libraries preparation and mRNA sequencing was conducted at the GeT‐Plage facility (INRAE Castanet‐Tolosan, France) using the Illumina TrueSeq Stranded mRNA kit, 2 × 150 base pair reads sequencing with S4 chemistry on a NovaSeq instrument. The reads were simultaneously aligned to the Arabidopsis Columbia‐0 reference genome Araport11 and the *S. sclerotiorum* strain 1980 v.2 genome, using the pipeline nf‐core/rnaseq v.3.12.0 described in doi: 10.5281/zenodo.1400710. Reads were normalized by calculated Counts Per Million for *A. thaliana* mapped reads. Genes with CPM ≤ 2 for 10 or more samples (out of 18) were considered low expressed and excluded from further analysis, leaving 12 892 expressed genes. Differential expression analysis was conducted using deseq2 package v.1.42.1 using the design = ~ replicate + genotype formula and contrasts between *nac42‐L* mutants and Col‐0, considering samples from temperate and Mediterranean acclimation separately. Genes with |LFC| ≥ 1 and adjusted value ≤ 0.01 were considered significant for differential expression. Gene Ontology enrichment analyses were performed using BINGO module 3.0.5 from Cytoscape 3.9.1, assessing over‐representation of BP gene ontology (GO) terms in our gene lists compared GO terms in the 12 892 expressed genes, using a hypergeometric test with *P*‐value cutoff of 0.05. Heatmaps were generated using the heatmap.2 packages in R 4.2.1. The HMM model NAC_tnt.AT3G12910_col_a_m1 for the unique DAP‐seq motif bound by AT3G12910 was obtained from the Plant Cistrome Database (http://neomorph.salk.edu/dap_web/) (O'Malley *et al*., 2016). *Arabidopsis thaliana* genes harboring the corresponding motif were identified using Fimo v.5.5.5 (Grant *et al*., 2011) using the sequence 1 kbp upstream of the translation start site from the Araport11 annotation with 1E‐4 as *P*‐value threshold, both strands scanning and NRDB frequencies as a background model.

## Results

### Mediterranean‐like acclimation impairs the QDR of several *A. thaliana* accessions to *S. sclerotiorum*

**Fig. 1:**
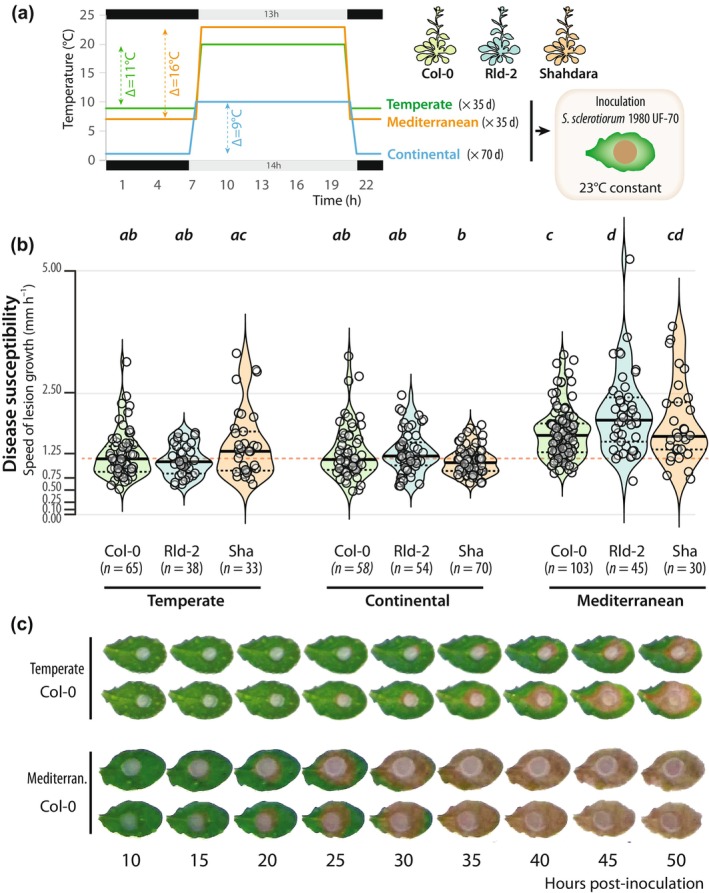
Effect of three distinct pre‐infection acclimation conditions on *Arabidopsis thaliana* quantitative disease resistance (QDR) to *Sclerotinia sclerotiorum*. (a) Experimental design showing acclimation and inoculation phases. Daylength, day and night temperatures typical of temperate, continental and Mediterranean acclimation conditions define the three acclimation conditions used in this work. (b) QDR phenotype in response to *S. sclerotiorum* infection as a function of acclimation and genotype. Each experiment was repeated at least three times and the significance of the results was assessed by an ANOVA followed by a Tukey HSD test, with significance groups labeled by letters. Lines show first quartile, median and third quartile values. (c) Representative symptoms of Col‐0 plants between 10 and 50 h post inoculation by *S. sclerotiorum* on leaves harvested on plants acclimated in temperate and Mediterranean (Mediterran.) conditions.

To determine the effect of acclimation on *A. thaliana* QDR, we analyzed phenotypic variation of three *A. thaliana* accessions after growth in three simulated climates. We selected accessions Col‐0, Rld‐2 and Shahdara (Sha) to span the geographic diversity of the native Eurasian and North African range of the species (Alonso‐Blanco *et al*., 2016) and genetic differentiation (Lian *et al*., 2024) while maintaining experimental feasibility. For acclimation, plants were grown under day length, day and night temperatures corresponding to the 30‐yr average for the month of April in areas with a temperate (Cfa, 20°C : 9°C, day : night), continental (Dfb, 11°C : 1°C, day : night) and Mediterranean (Csa, 23°C : 7°C, day : night) climates (Figs 1a, S1). These correspond to climates in the distribution range of *A. thaliana* with major projected area variation by the end of this century (Peel *et al*., 2007; Alonso‐Blanco *et al*., 2016; Cui *et al*., 2021). We will refer to these conditions as temperate, continental and Mediterranean acclimation to reflect differences from true climate conditions found in nature. Plants were grown for 35 d under temperate and Mediterranean acclimation, corresponding to 13 405 and 13 930°C d, and for 70 d under continental acclimation, corresponding to 11 480°C d, before inoculation with *S. sclerotiorum* under infection‐conducive conditions. Plants were not flowering at the time of inoculation (Fig. S2). Quantification of effect size for morphological measurements indicated that at the time of inoculation, continental acclimation had a large effect on leaf roundness and Mediterranean acclimation had a medium effect on leaf area (Fig. S2). Plant QDR was assessed using time‐resolved automated phenotyping as the characteristic time for disease lesion increase (Barbacci *et al*., 2020). After temperate acclimation, all accessions had similar QDR levels with only a slightly higher QDR (9% average) for Rld‐2 and a slightly lower QDR for Sha (+18% average) compared to Col‐0 (Fig. 1b,c; Table S2). These phenotypes were not significantly altered upon continental acclimation. Mediterranean acclimation rendered all accessions significantly more susceptible, with an average QDR decrease by 29% for Sha, 37% for Col‐0 and 86% for Rld‐2 as compared to temperate acclimation. These results show that both genotype and acclimation affect the susceptibility of *A. thaliana* to *S. sclerotiorum* and that, regardless of genotype, the warmest acclimation (Mediterranean) caused the most significant loss of QDR.

### Transcriptome profiling highlights pathogen response reprogramming following acclimation

To study the molecular bases of QDR acclimation, we performed a global transcriptome analysis of *A. thaliana* accessions Col‐0, Rld‐2 and Sha grown under temperate, continental and Mediterranean acclimation, followed or not by *S. sclerotiorum* inoculation. We found 17 137 nuclear‐encoded genes with sufficient coverage (Table S3) for further analyses. Differential analysis revealed limited overlap in the transcriptomic response to acclimation in non‐inoculated plants (|LFC| ≥ 2 and Bonferroni‐adjusted *P*‐val < 1E‐4, Fig. S3; Tables S4, S5). In the absence of a pathogen, Mediterranean acclimation activated pathways such as response to jasmonic acid and to water deprivation, while continental acclimation activated response to chitin and to SA. Genes related to the regulation of transcription were differentially expressed upon Mediterranean and continental acclimation relative to temperate acclimation.

**Fig. 2:**
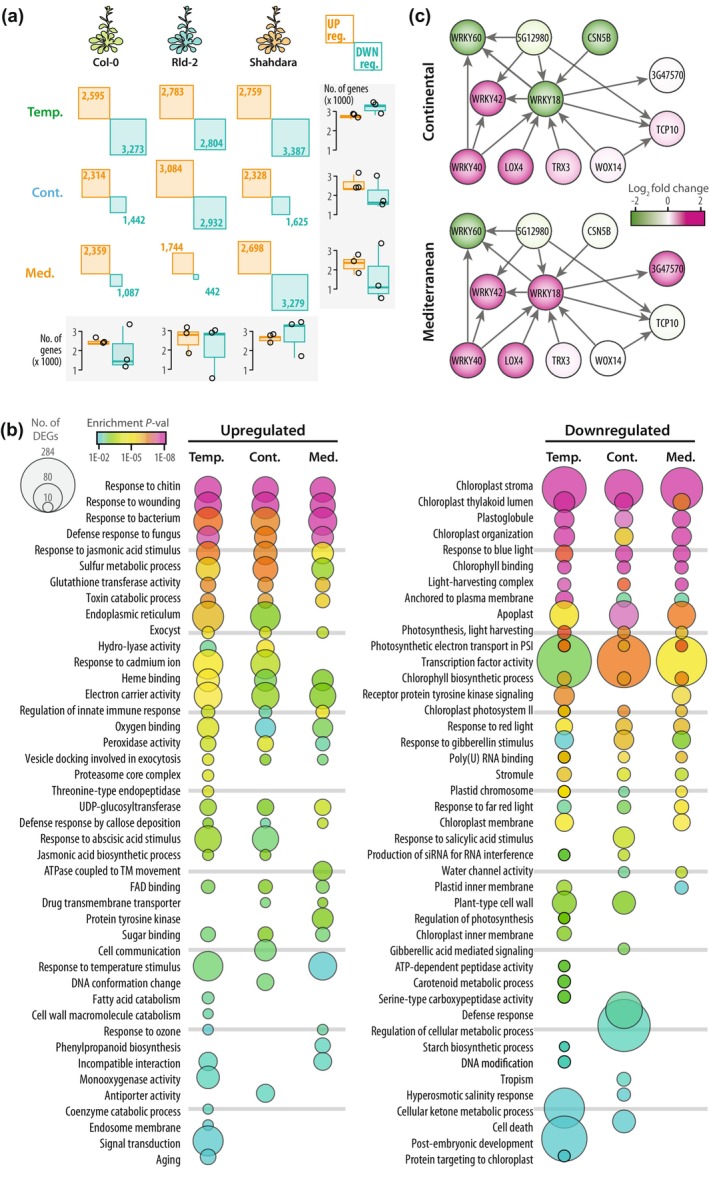
Global gene expression profiling of *Arabidopsis thaliana* plants inoculated by the fungal pathogen *Sclerotinia sclerotiorum* following temperate, continental and Mediterranean acclimation. (a) Number of differentially expressed genes (DEGs) upregulated (yellow) and downregulated (blue) 48 h post inoculation by *S. sclerotiorum* in each of three plant genotypes and three acclimation conditions. Box plots show the distribution of DEG number per genotype (columns) and acclimation (rows), first and third quartiles (box), median (thick line) and the most dispersed values within 1.5 times the interquartile range (whiskers) are shown. (b) Gene ontologies (GOs) enriched among upregulated (left) and downregulated (right) DEGs upon pathogen inoculation according to plant acclimation. Circles are sized according to the number of DEGs per GO and colored according to the enrichment *P*‐value (Hypergeometric test with Benjamini‐Hochberg correction). (c) Subset of the gene and protein network connected to the transcription factor WRKY18 through co‐expression and protein–protein interaction relationships. Circles representing genes are colored according to their expression (Log_2_ fold change upon pathogen inoculation) after continental (top) and Mediterranean (bottom) acclimation.

To identify genes responsive to infection, we performed a differential expression analysis using non‐inoculated plants as reference in each of nine conditions (three acclimation regimes, times three plant genotypes) and identified 9580 differentially expressed genes (DEGs) upon inoculation (Figs 2a, S4, S5; Table S6). The number of upregulated genes ranged from 1744 (Rld‐2 Mediterranean acclimation) to 3084 (Rld‐2 continental acclimation), and the number of downregulated genes ranged from 442 (Rld‐2 Mediterranean acclimation) to 3387 (Sha temperate acclimation). Genes differentially expressed after all three acclimation regimes represented 33.4 ± 10.1% of DEGs and genes differentially expressed specifically after one acclimation regime represented 35.1 ± 6.3% of DEGs (Fig. S5), revealing a substantial effect of acclimation on the subsequent transcriptional response to pathogen inoculation.

To summarize changes to *S. sclerotiorum* responses following acclimation, we performed GO enrichment analyses in DEGs upon inoculation (Fig. 2b; Table S7). We compared the acclimation regimes considering genes differentially expressed in any of the three *A. thaliana* accessions and using the 17 137 expressed genes as a reference set. Low‐level GOs significantly enriched in DEGs ranged from 24 (DEGs downregulated following Mediterranean acclimation) to 36 (DEGs upregulated following temperate acclimation). 44.3% of GOs were enriched in DEGs following all three acclimation regimes and 36.4% of GOs were enriched in DEGs only following one specific acclimation regime. For instance, among upregulated DEGs, ontologies ‘phenylpropanoid biosynthesis’ and ‘protein tyrosine kinase’ were only enriched following Mediterranean acclimation, ‘cell communication’ and ‘DNA conformation change’ were only enriched following continental acclimation and ‘monooxygenase activity’ and ‘threonine‐type endopeptidase’ were only enriched following temperate acclimation. With 284 downregulated DEGs following temperate acclimation, ‘transcription factor activity’ was the most abundant low‐level GO enriched in DEGs. To illustrate the reprogramming of transcription factor networks following acclimation, we used public co‐expression and protein interaction data to reconstruct the network immediately related to *WRKY18* (*AT4G31800*) and study its behavior after acclimation (Figs 2c, S5C). While *WRKY18* was downregulated after continental acclimation (LFC ‐2.14), it was upregulated after Mediterranean acclimation (LFC 2.40), possibly as a result of variations to *CSN5B* (*AT1G71230*) and *TRX3* (*AT5G42980*) expression, located upstream in the network. Downstream of *WRKY18*, while *TCP10* (*AT2G31070*) was more induced than *AT3G47570* after continental acclimation (LFC 0.91 and 0.13, respectively), this pattern was reversed after Mediterranean acclimation (LFC −0.25 and 3.45, respectively).

### Identification of candidate acclimation‐proof regulators of QDR

**Fig. 3:**
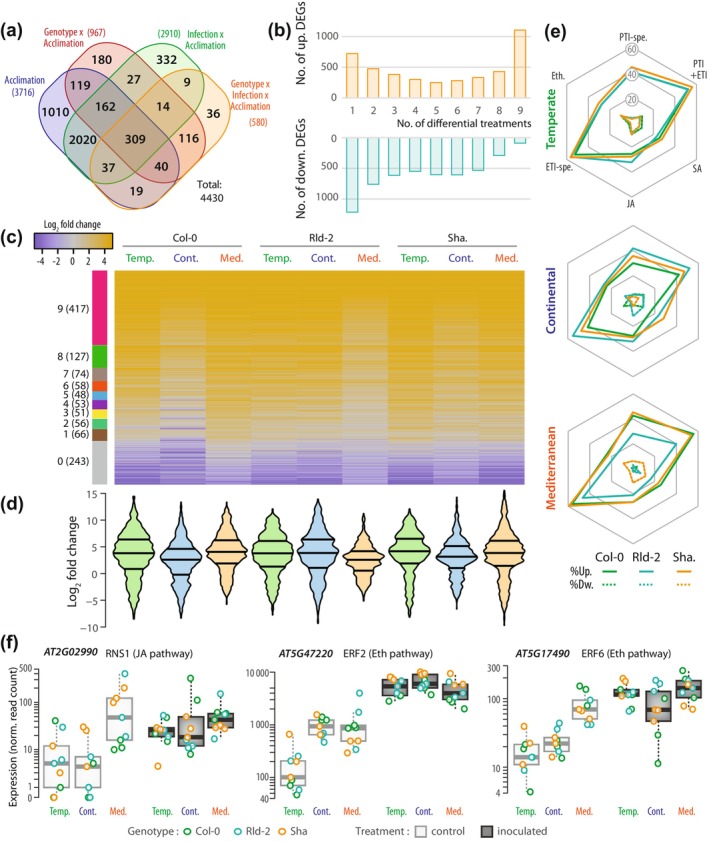
Effect of acclimation on the regulation of *Arabidopsis thaliana* genes responsive to *Sclerotinia sclerotiorum* inoculation. (a) Distribution of acclimation‐dependent DEGs identified by ANOVA according to the factors explaining gene expression variance. (b) Number of up‐ and downregulated DEGs according to the number of treatments causing differential expression, out of nine possible (3 genotypes × 3 acclimations). (c) Expression variation (log_2_ fold change) upon pathogen inoculation for 1193 immunity‐related genes in three *A. thaliana* accessions following temperate (Temp.), continental (Cont.) and Mediterranean (Med.) acclimation. (d) Distribution of log_2_ fold change upon pathogen inoculation for 1193 immunity‐related genes. Treatments are ordered as in (c). The middle line of violin plots shows the median value and the outer lines are the first and third quartiles. (e) Proportion of genes (%) from six major immunity pathways upregulated (Up., plain lines) and downregulated (Dw., dotted lines) upon pathogen inoculation in each *A. thaliana* genotype according to acclimation. (f) Expression in normalized read count of three selected genes from the JA and Eth pathways, highlighting specific regulation following Mediterranean acclimation. Boxplots show median (plain line), first and third quartile (box) and 1.5 times the interquartile range (whiskers). Eth, ethylene; ETI, effector‐triggered immunity; JA, jasmonic acid; PTI, pathogen‐associated molecular pattern‐triggered immunity; SA, salicylic acid; spe., specific.

With an aim to identify promising genetic targets supporting acclimation‐proof immunity, we focused on genes weakly sensitive to acclimation and robustly induced upon pathogen inoculation. First, we performed an analysis of variance on all expressed genes and classified the 9580 DEGs according to which of the plant genotype, infection status, acclimation and their interactions contributed the most to expression variation for each gene. As expected, infection contributed significantly (Benjamini–Hochberg corrected *P*‐val ≤ 2E‐03) to the expression variance for 8026 DEGs (83.8%). Genotype and acclimation contributed significantly to the expression variance for 3211 and 3786 DEGs (33.5% and 39.5%), respectively (Figs 3a, S6; Table S8). The median acclimation‐related variance (sum of variance for acclimation factor and interactions between acclimation and any other factor) was 24.5% for all expressed genes and 22.1% for DEGs. Among genes responsive to infection, 4518 (47.1% of DEGs) had an expression significantly dependent on either acclimation alone or interaction between acclimation and any other factor, with a median acclimation‐related variance 31.1%.

Second, we classified DEGs according to the robustness of their regulation upon pathogen inoculation. Downregulated genes showed a relative degree of specificity with 1212 genes (23%) unique to one genotype‐acclimation pair, and only 89 (1.7%) genes were differentially expressed in all nine genotype‐acclimation conditions tested (Fig. 3b; Table S4). Upregulated genes showed higher robustness with 1105 genes (26.8%) differential in all nine genotype‐acclimation conditions (Table S4). These 1105 genes are promising candidates to contribute to a robust acclimation‐proof pathogen response. Among those, 776 had an expression variance significantly influenced by inoculation but not by other factors (*P*‐val threshold 1E‐05, median acclimation‐related variance 8.9%, Fig. S7).

Third, we collected a list of 1193 genes differentially expressed upon *S. sclerotiorum* inoculation and associated with *A. thaliana* immune responses in (Hatsugai *et al*., 2017; Delplace *et al*., 2020; Bjornson *et al*., 2021), and studied their regulation (Fig. 3c; Table S9). Thirty‐five percent (417 genes) were upregulated by *S. sclerotiorum* in all accessions and all acclimation scenarios, while 20% (243 genes) were downregulated in all samples, probably representing immune responses irrelevant to interaction with *S. sclerotiorum*. To document the impact of acclimation on the expression of the 1193 immune‐related genes, we analyzed their distribution of LFC upon inoculation across samples (Fig. 3d). In Sha., the effect of acclimation was negligible (Cohen's *d* < 0.2), and it ranged from negligible to small (Cohen's *d* < 0.5) in Col‐0 and Rld‐2, indicating a relative stability of immune genes expression according to acclimation. Except in Rld‐2, Mediterranean acclimation did not lead to an average decrease in immune genes LFC upon inoculation. In Col‐0 and Rld‐2, Mediterranean acclimation resulted in a reduced LFC amplitude with an interquartile range reduced to respectively 77.5% and 74.8% of its amplitude, respectively, after temperate acclimation. We then analyzed the proportion of genes up‐ and downregulated upon *S. sclerotiorum* inoculation from six major immune pathways (Fig. 3e). Pathogen‐associated molecular pattern‐triggered immunity (PTI) and effector‐triggered immunity (ETI) responses were strongly upregulated and not significantly altered by acclimation, while fewer genes from the phytohormone signaling pathways (SA, JA and ethylene) were upregulated after Mediterranean acclimation. In several cases, this was due to constitutive activation in non‐inoculated plants following Mediterranean acclimation (Fig. 3f). Compared to temperate acclimation, continental and Mediterranean acclimation amplified differences across accessions in the number of immune genes differentially regulated.

Together, these analyses provide a list of known immune genes and novel *S. sclerotiorum*‐responsive genes, the expression of which is not significantly altered by acclimation.

### A community of upregulated genes associates with acclimation‐proof QDR

**Fig. 4:**
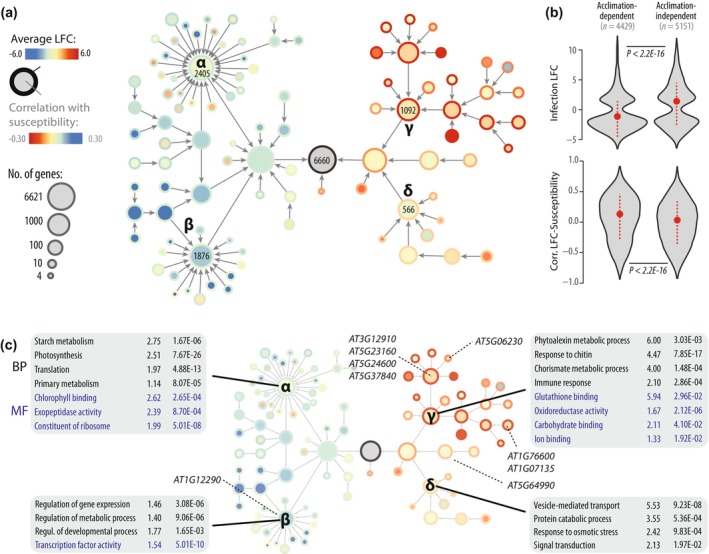
Hierarchical co‐expression network and properties of gene communities for genes responsive to *Sclerotinia sclerotiorum* inoculation (a) Hierarchical network of genes misregulated by *S. sclerotiorum* infection identified through differential and variance analyses. Nodes represent gene communities sized by the number of differentially expressed genes (DEGs) they contain, fill color corresponding to the average correlation between gene expression and plant susceptibility, border color corresponding to the average log_2_ fold change (LFC) upon inoculation. Four major communities are labeled and their number of genes indicated. (b) Distribution of infection LFC and correlation between LFC and susceptibility phenotype for acclimation‐dependent and independent DEGs. Violin plots show a Gaussian kernel, median (dot) and SD (dotted lines). (c) Gene ontology (GO) enrichment in the four major gene communities identified based on a co‐expression network of genes misregulated by *S. sclerotiorum* infection. Communities α, β, γ, δ are labeled on the hierarchical network shown with the same layout as in (a). A selection of the most enriched biological processes (BP, black) and molecular functions (MF, blue) GOs are labeled, with enrichment fold and adjusted *P*‐value relative to the *Arabidopsis thaliana* genome indicated. Genes analyzed through mutant phenotyping are labeled according to their position in the network.

To document the relationship between acclimation‐proof DEGs and other transcriptional responses to *S. sclerotiorum* inoculation, we built a gene co‐expression hierarchical network with genes modulated by inoculation both in the differential and ANOVA analyses. For this, we used normalized read counts to calculate the Spearman rank correlation coefficient for all pairwise gene comparisons across our 54 RNA‐seq samples. Highly co‐expressed gene pairs were grouped into hierarchical gene communities using the HiDeF algorithm (Zheng *et al*., 2021). 6620 genes were included in communities of at least four genes (Fig. 4a; Dataset S1). Four major top‐level communities (labeled α to δ in Fig. 4a) encompassed 5933 genes (89.6% of the network). On average, communities α and β included genes with expression anticorrelated with QDR (putative susceptibility factors, Table S10), frequently acclimation‐dependent and downregulated upon *S. sclerotiorum* inoculation and upon heat stress (37°C for 3 h (Guo *et al*., 2022)). By contrast, genes from communities γ and δ had expression correlated with QDR, frequently acclimation‐independent, upregulated upon *S. sclerotiorum* inoculation and heat stress (Figs 4a, S7). Of the 1105 genes upregulated in all conditions we identified previously, 851 fell in community γ and 38 in community δ; none were in communities α and β.

Throughout the network, genes acclimation‐independent tended to be more induced upon inoculation (median LFC 0.70) and less associated with quantitative disease susceptibility (12.3% with Pearson correlation to susceptibility > 0.5) than genes dependent on acclimation (median LFC −1.56, 19.2% with Pearson correlation to susceptibility > 0.5) (Fig. 4b). This suggests that genes upregulated upon inoculation are more likely to contribute to robust acclimation‐proof pathogen response than downregulated genes. In agreement, genes upregulated by inoculation were 1.48‐fold more abundant among acclimation‐independent genes (52.6%) than acclimation‐independent genes (35.5%).

To get insights into the role of DEGs in *A. thaliana* QDR against *S. sclerotiorum*, we first analyzed Gene Ontologies (GO) enriched in each of the four major top‐level gene communities from our hierarchical network, relative to the rest of the network (Fig. 4c; Table S11). Community α was enriched in 96 biological processes (BP) and 9 molecular functions (MF) GO, with ‘Starch metabolism’, ‘Photosynthesis’, ‘Translation’, ‘Primary metabolism’, ‘Chlorophyll binding’, ‘Exopeptidase activity’ and ‘Constituent of ribosome’ among the most enriched, reflecting a general downregulation of energetic functions of the plant cell during infection. Community β was enriched in 18 BP and 4 MF GOs with ‘Regulation of gene expression’, ‘Regulation of metabolic process’, ‘Regulation of developmental process’ and ‘Transcription factor activity’ among the most enriched. Community γ was enriched in 68 BP and 22 MF GOs, with ‘Phytoalexin metabolic process’, ‘Response to chitin’, ‘Chorismate metabolic process’, ‘Immune response’, ‘Glutathione binding’, ‘Oxidoreductase activity’, ‘Carbohydrate binding’ and ‘Ion binding’ among the most enriched, reflecting the probable involvement of genes from this community in QDR. Finally, community δ was enriched in 40 BP and 10 MF GOs, with ‘Vesicle‐mediated transport’, ‘Protein catabolic process’, ‘Response to osmotic stress’ and ‘Signal transduction’ among the most enriched, consistent with a role in stress response. These ontology enrichments point toward community γ as a promising source of QDR‐related genes.

### Mediterranean‐like acclimation increases QDR of several susceptible mutant genotypes

**Fig. 5:**
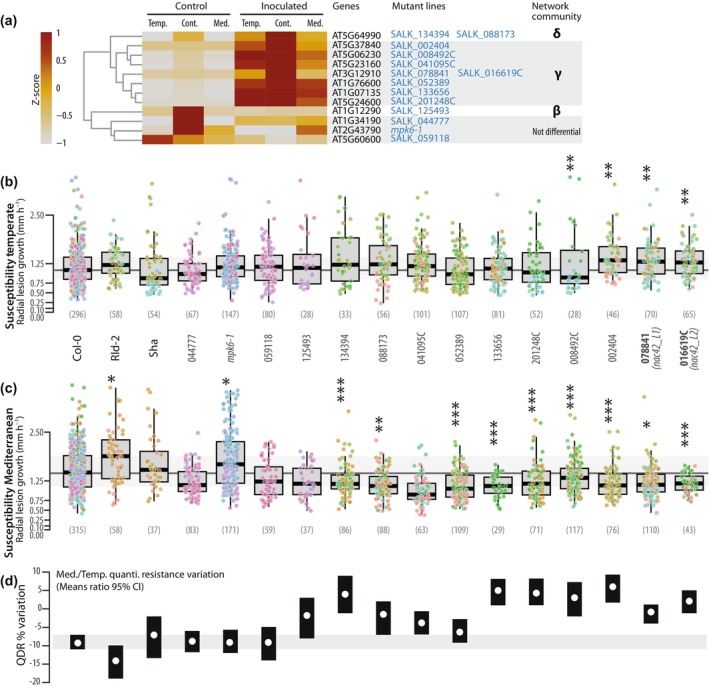
Quantitative disease resistance phenotypes of *Arabidopsis thaliana* mutant genotypes following temperate and Mediterranean acclimation. (a) Relative expression of selected genes in control and inoculated samples following temperate (Temp.), continental (Cont.) and Mediterranean (Med.) acclimation. The table indicates corresponding mutant lines and gene network communities. Genes not differentially expressed upon *Sclerotinia sclerotiorum* inoculation are not part of the co‐expression network and are provided as controls. (b, c) Disease susceptibility phenotype of natural accessions and mutant plants following temperate acclimation (b) or Mediterranean acclimation (c) inoculated by *S. sclerotiorum*. Mutant lines are designated by their 5‐digit SALK line code. Boxplots show first and third quartiles (box), median (thick line) and the most dispersed values within 1.5 times the interquartile range (whiskers). Colors of the data points indicate independent inoculation experiments. Leaves from *n* = 29 to 315 plants were tested for each genotype. Significance of the difference from Col‐0 wild‐type was assessed by a Kruskal–Wallis test followed by Benjamini‐Hochberg correction for multiple testing (***, *P* < 0.01; **, *P* < 0.05; *, *P* < 0.1). (d) Percentage variation in disease resistance phenotype between temperate and Mediterranean‐acclimated plants, calculated as −100 × (Med./Temp. susceptibility means ratio − 1). Bars show 95% estimated confidence interval of the mean ratio.

To study the role of DEGs in QDR against *S. sclerotiorum*, we analyzed the phenotype of 14 mutant genotypes in the Col‐0 background, including mutations in 11 distinct genes (Fig. 5a). With an aim to discover new genes associated with acclimation‐proof QDR, we focused on genotypes including mutations in genes from community γ that were not previously associated with plant immunity (Table S12). For comparison purposes, we included genotypes with a mutation in *AT1G12290* from community β, *AT5G64990* from community δ and *AT1G34190*, *MPK6* (*AT2G43790*) and *AT5G60600*, not differentially expressed in our RNA‐seq experiment. The natural accessions Col‐0, Rld‐2 and Sha were used as references. The growth of these genotypes was not drastically altered by temperate and Mediterranean acclimation (Fig. S8). After temperate acclimation (Fig. 5b), four mutant genotypes were significantly more susceptible than the Col‐0 wild‐type, including mutations in *AT5G06230*, *AT5G37840* and the two mutant alleles in *AT3G12910* from community γ. After Mediterranean acclimation (Fig. 5c; Table S13), all three natural accessions were more susceptible than after temperate acclimation, consistent with our previous set of experiments (Fig. 1b). Rld‐2 was more strongly affected by Mediterranean acclimation and became significantly more susceptible than Col‐0 in these conditions. To our surprise, *mpk6‐1* was the only mutant significantly more susceptible than Col‐0 after Mediterranean acclimation. Nine genotypes were more resistant than wild‐type after Mediterranean acclimation, including mutations in *AT1G76600*, *AT1G07135*, *AT5G24600*, *AT5G06230*, *AT3G12910* (two alleles) and *AT5G37840* from community γ and *AT5G64990* (two alleles) from community δ. While natural accessions had their QDR phenotype reduced by *c*. 10% on average after Mediterranean compared to temperate acclimation (Fig. 5d), only four mutant genotypes showed > 5% QDR reduction, including three with mutations in genes not differentially expressed upon inoculation (*AT1G34190*, *AT2G43790* and *AT5G60600*) and one from community γ (*AT1G76600*). By contrast, seven genotypes showed increased QDR after Mediterranean compared to temperate acclimation (95% confidence interval for means ratio extending > 0), including two lines with mutations in *AT3G12910* and in *AT5G64990*.

Although causal alleles remain to be unambiguously determined, these results point toward community γ as a reservoir of genes contributing to QDR against *S. sclerotiorum* after temperate acclimation. Genotypes including mutations in several genes from community γ were more resistant than wild‐type after Mediterranean acclimation, indicating that they include mutations in genes acting as susceptibility factors in these conditions. Mutations in *AT3G12910* and *AT5G64990* were allelic in two genotypes, suggesting that these genes play a key role in the phenotypes we observed. These two genes were upregulated in all nine conditions tested (LFC 2.41–4.61 and 4.74–7.83 for AT5G64990 and AT3G12910, respectively). Remarkably, AT3G12910 would be classified as an immunity factor in temperate‐acclimated plants but as a susceptibility factor in Mediterranean‐acclimated plants.

### Acclimation shifts the repertoire of NAC42‐L target genes upon *S. sclerotiorum* inoculation

**Fig. 6:**
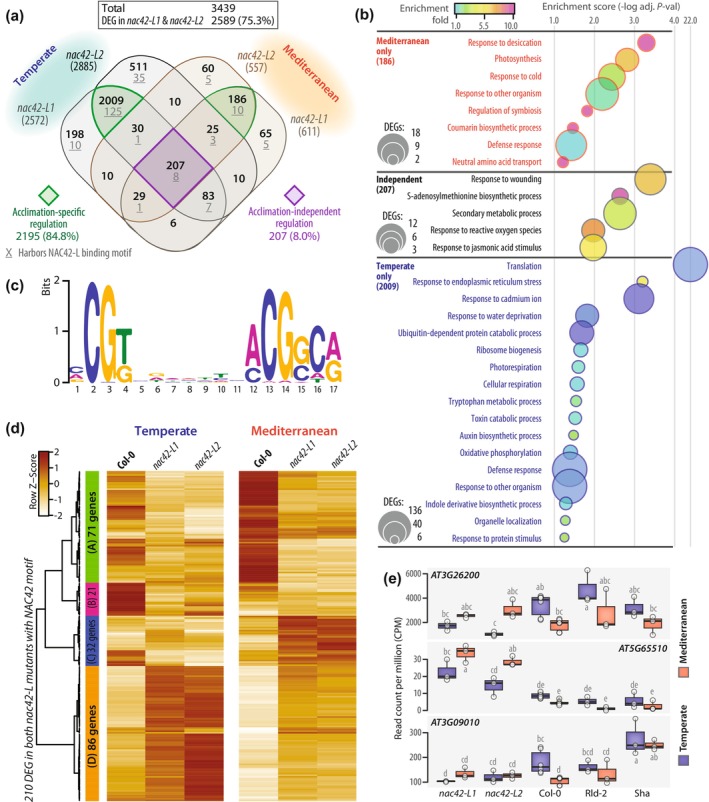
Effect of temperate and Mediterranean acclimation on the regulation of gene expression by the transcription factor AtNAC42‐L upon *Sclerotinia sclerotiorum* inoculation. (a) Distribution of differentially expressed genes (DEGs) in *nac42‐L* mutants relative to Col‐0 at 24 h post inoculation by *S. sclerotiorum* following temperate and Mediterranean acclimation. Genes differentially expressed in the two mutant lines representing 75.3% of all DEGs are highlighted that showed either acclimation specific regulation (green sectors) or acclimation‐independent regulation (purple sector). The number of DEGs harboring an NAC42‐L binding motif in their promoter is indicated underlined. (b) Low‐level biological process gene ontologies (GOs) significantly enriched among DEGs common to the two *nac42‐L* mutant lines for Mediterranean‐specific DEGs, acclimation‐independent DEGs and temperate‐specific DEGs. Bubbles are sized according to the number DEGs with each GO and colored according to enrichment fold relative to all expressed genes. GOs are ranked in increasing order of enrichment *P*‐value. (c) Sequence logo of the promoter motif bound by NAC42‐L according to DAP‐seq data. (d) Heatmap showing the relative expression of DEGs common to the two nac42‐L mutants and harboring an NAC42‐L binding motif in their promoter. Four gene clusters designated A to D are labeled, with the number of genes in each indicated. (e) Expression in read count per million of three selected NAC42‐L target genes in inoculated samples, highlighting contrasted effects of *nac42‐L* mutation following temperate and Mediterranean acclimation. Boxplots show median (plain line), first and third quartile (box) and 1.5 times the interquartile range (whiskers). Letters indicate Tukey HSD significance groups.

*AT3G12910* encodes a member of the NAC family of transcription factors that includes several regulators of pathogen and abiotic stress response (Nuruzzaman *et al*., 2013). Its closest homolog in the *A. thaliana* genome is *NAC42/JUNGBRUNNEN1* (*AT2G43000*) (Ooka *et al*., 2003), we will thus refer to *AT3G12910* as *NAC42‐Like* (*NAC42‐L*) hereafter. *NAC42‐L* is strongly induced upon *S. sclerotiorum* inoculation both in plants temperate‐ (LFC 7.8 *P*‐adj. 3E‐08 in Col‐0) and Mediterranean‐acclimated (LFC 7.5 *P*‐adj. 8E‐22 in Col‐0). Yet two mutant alleles of *NAC42‐L* resulted in lower QDR in temperate‐acclimated plants but enhanced QDR in Mediterranean‐acclimated plants (Figs 5, S9). To study how acclimation alters the activity of NAC42‐L at the molecular level in inoculated plants, we analyzed the expression of its target genes upon infection in temperate‐ and Mediterranean‐acclimated plants. For this, we collected RNA‐seq reads from Col‐0, *nac42‐L1* (SALK_016619C) and *nac42‐L2* (SALK_078841) mutant plants 24 h post inoculation by *S. sclerotiorum* after temperate and Mediterranean acclimation. We identified 12 892 genes expressed to sufficient level across these samples (CPM > 2 in 8 or more samples) and performed a differential expression analysis comparing inoculated *nac42‐L* mutants with Col‐0 in each acclimation regime (Fig. 6a). This revealed 3439 DEGs in total (Tables S14, S15), among which 2589 (75.3%) were shared between *nac42‐L1* and *nac42‐L2*, supporting a major role for *NAC42‐L* disruption in the observed regulation patterns. There were 207 DEGs common to the two *nac42‐L* mutant lines and the two acclimation treatments, representing NAC42‐L acclimation‐independent target repertoire (8.0% of DEGs shared between *nac42‐L1* and *nac42‐L2*). Out of 2589 DEGs shared between *nac42‐L1* and *nac42‐L2*, 2195 (84.8%) were differential between Col‐0 and *nac42‐L* mutants following one of the acclimation regimes only (2009 and 186 after temperate and Mediterranean acclimation, respectively). This reveals a significant switch in the regulation of NAC42‐L target genes upon infection according to acclimation.

To get insights into the BP affected by a switch in NAC42‐L target genes repertoire, we analyzed ontologies enriched among DEGs in *nac42‐L1* and *nac42‐L2* compared to Col‐0 (Fig. 6b; Table S16). Eight terminal ontologies were enriched among the 186 NAC42‐L putative targets specific to Mediterranean acclimation, including response to desiccation, response to cold and defense response. Five terminal ontologies were enriched among the 207 NAC42‐L acclimation‐independent targets, including response to wounding, S‐adenosylmethionine biosynthesis, response to reactive oxygen species and response to jasmonic acid. These GOs are all relevant to plant defense response, suggesting that NAC42‐L retains the ability to regulate plant immunity to some degree in diverse acclimation conditions. Finally, 17 terminal ontologies were enriched among the 2009 NAC42‐L putative targets specific to temperate acclimation. Four of these (defense response, response to other organisms, response to water deprivation and photorespiration) were identical or similar to GOs enriched among NAC42‐L putative targets specific to Mediterranean acclimation, suggesting that different genes involved in similar processes may be targeted by NAC42‐L according to acclimation. Other GOs were unique to NAC42‐L, putative targets specific to temperate acclimation, such as protein translation, ubiquitination and toxin catabolic process, highlighting distinct processes putatively regulated by NAC42‐L according to acclimation.

To predict direct targets of NAC42‐L among genes misregulated in nac42‐L mutants, we searched for the NAC42‐L DNA binding motif determined by (O'Malley *et al*., 2016) in the promoter of *A. thaliana* genes (Fig. 6c). This identified 2276 potential NAC‐L binding sites in 1795 different gene promoters, with a maximum of 5 binding sites per promoter (Table S17). Among NAC42‐L putative targets identified by RNA‐seq in nac42‐L mutants, 210 harbored NAC42‐L binding motifs in their promoter, representing possible direct targets. Among those, 190 (90.5%) were uniquely differential in *nac42‐L* mutants compared to Col‐0 following one acclimation regime (Fig. 6a). Hierarchical clustering based on the expression of the 210 predicted direct targets of NAC42‐L distinguished four major clusters (Fig. 6d; Table S18). Seventy‐one genes (cluster A) were generally less expressed in *nac42‐L* mutants than in Col‐0 following both temperate and Mediterranean acclimation. Conversely, 86 genes (cluster D) were more expressed in *nac42‐L* mutants than in Col‐0 with the two acclimation treatments. For instance, *AT5G51290*, encoding the ACCELERATED CELL DEATH ceramide kinase ACD5, was *c*. 2.9‐fold more expressed in *nac42‐l* mutants than in natural accessions (Fig. S10). Interestingly, Mediterranean acclimation increased *AT5G65510* expression in *nac42‐l* mutants relative to temperate acclimation, but the opposite pattern was observed in natural accessions. Cluster B included 21 genes strongly downregulated in *nac42‐L* mutants compared to Col‐0 following temperate acclimation only. For instance, *AT3G26200* encoding the cytochrome P450 CYP71B22 showed a trend for lower expression in *nac42‐l* mutants than in Col‐0, Rld‐2 and Sha following temperate acclimation, but a slight trend for higher expression in *nac42‐l* mutants than in natural accession following Mediterranean acclimation (Fig. 6e). Cluster C included 32 genes strongly upregulated in *nac42‐L* mutants compared to Col‐0 following Mediterranean acclimation. One example is *AT5G65510* encoding the AP2/ERF transcription factor AINTEGUMENTA‐like 7, which was more strongly expressed in *nac42‐l* mutants than in Col‐0, Rld‐2 and Sha following Mediterranean acclimation and to a lesser extent after temperate acclimation (Figs 6e, S10). While drastic changes to the regulation of genes from clusters B and C are observed following acclimation, quantitative changes also affect the regulation of genes from clusters A and D, explaining the prevalence of acclimation‐specific regulation in NAC42‐L direct predicted targets revealed by differential analysis. For instance, *AT3G09010* (Cluster A), encoding an uncharacterized protein kinase, was less expressed in *nac42‐l* plants than in Col‐0 following temperate acclimation but not following Mediterranean acclimation (Fig. 6e). Therefore, in spite of being consistently upregulated by inoculation in all acclimation scenarios, NAC42‐L drives acclimation‐dependent responses to pathogens through acclimation‐dependent sets of target genes. Together, these results suggest that acclimation alters the contribution of NAC42‐L to QDR by switching the repertoire of genes regulated by this transcription factor.

## Discussion

Phenotypic plasticity, a component of acclimation, allows plant species to adjust to environmental conditions, together with adaptation through natural selection or migration to follow conditions to which they are adapted. Understanding the molecular mechanisms of acclimation is crucial for predicting changes in species distributions, community composition and crop productivity under climate change. In this work, we show that *A. thaliana* Mediterranean acclimation is detrimental for QDR to the fungus *S. sclerotiorum* and converts several genes that contribute positively to quantitative immunity following temperate acclimation into susceptibility factors. Mediterranean acclimation involves a shift in the repertoire of targets of the pathogen‐induced transcription factor NAC42‐like that may impair the regulation of quantitative immune responses.

Experiments in controlled conditions have been instrumental in unraveling complex stressor interactions through tightly controlled factorial experiments. These studies emphasized that the combined effects of various environmental stressors resulted in unique transcriptional changes distinct from individual stress responses (Sewelam *et al*., 2014; Zandalinas & Mittler, 2022). These interactions can be synergistic, where stressors amplify each other's negative effects, or antagonistic, where they dampen each other's impacts (Zarattini *et al*., 2021). Research on plant‐pathogen interactions under abiotic constraints often relies on long‐lasting stable temperature shifts, overlooking the complex acclimation processes plants undergo in response to gradual climatic shifts (Aoun *et al*., 2017; Desaint *et al*., 2021). Several studies investigated the effect of temperature acclimation by applying a stable temperature shift over a few days before a second stress application. For instance, growth of *A. thaliana* for 7 d at 4°C enhanced survival to freezing in a NPR1‐dependent manner (Olate *et al*., 2018), and 2 d growth at 30°C rendered plants more susceptible to the bacterial pathogen *Pseudomonas syringae* pv *tomato* DC3000 when inoculation is performed either at 23°C or 30°C (Huot *et al*., 2017). Nevertheless, the impact of day–night temperature cycles on subsequent stress response is rarely considered. We have chosen to approximate climate change scenarios by simulating 30‐yr day and night average temperatures and photoperiods representing three climates of the Köppen–Geiger classification (Peel *et al*., 2007). In our design, the duration of the infection can be considered as a short (30 and 48 h) de‐acclimation period under constant 23°C temperature. To control for this in differential gene expression analyses, we harvested RNAs simultaneously from inoculated and non‐inoculated plants. Since the 1980s, Mediterranean climates with dry summer (Cs) have gradually replaced areas with temperate climate (Cf) (Cui *et al*., 2021). Predictions suggest that the Mediterranean (Csa) climate may replace a portion of the continental (Df, Dw, Ds) climates by the end of the century (Beck *et al*., 2018; Cui *et al*., 2021). Significant poleward shifts were observed for temperate (C), continental (D) and polar (E) climates with averages of 35.4, 16.2 and 12.6 km decennia^−1^ (0.32, 0.15 and 0.11° latitude decennia^−1^ respectively) and are expected to accelerate in the coming decades (Chan & Wu, 2015; Cui *et al*., 2021). The continental‐temperate‐Mediterranean sequence therefore reflects the predicted poleward shift of climate zones and associated changes in temperature and day length. Our work revealed a significant loss of QDR upon Mediterranean acclimation in multiple *A. thaliana* accessions, although the daily average temperature was only 0.7°C higher under Mediterranean acclimation (average 15.67°C) than under temperate acclimation (average 14.96°C). Plants sense daily and annual temperature fluctuations and changes to the photoperiod to adapt their reproduction and development (Ormancey & Qüesta, 2026), which may directly affect disease resistance (Lyons *et al*., 2015; Fabian *et al*., 2023). For instance, Arabidopsis plants exposed to repetitive heat, cold or salt stress show enhanced resistance to bacterial pathogens compared to plants maintained under stable conditions (Singh *et al*., 2014). In our study, we did not detect major alterations to clock genes expression due to acclimation (Fig. S2D), but downstream diel transcriptional entrainment effects may vary according to cell type (Redmond *et al*., 2025). Besides, nighttime temperatures may trigger cold‐enhanced immunity (Li *et al*., 2024). Nighttime temperatures and diel rhythms may also be linked since temperatures below 7°C trigger an attenuation of oscillation for diel rhythmic genes in *Arabidopsis helleri* (Muranaka *et al*., 2025). Given the current data, we cannot determine whether the observed phenotypic differences are attributable to daytime temperatures, nighttime temperatures, the photoperiod, or a combination of these factors, and whether they would be observed *in situ*. Nevertheless, our findings indicate that temperatures and daylength typical of April conditions in the Mediterranean climate zone, expected to expand by the end of the century due to global warming, are detrimental to QDR against *Sclerotinia* diseases. Combined with episodes of high humidity conducive to infection, global warming may therefore increase the incidence of these plant diseases.

High genetic variation in natural populations enhances their ability to withstand and adapt to new biotic and abiotic environmental changes, including climate change (Van Kleunen & Fischer, 2005; Nicotra *et al*., 2010). This genetic variation partly determines the capacity of plants to sense environmental changes and generate plastic responses. For instance, *cis*‐regulatory and epigenetic variation at the *FLOWERING LOCUS C* floral repressor regulating vernalization can aid plant populations in adapting to temperature fluctuations (Hepworth *et al*., 2020). Yet, the role of selection and whether gene expression plasticity facilitates or hinders adaptation remains a matter of debate (Levis & Pfennig, 2016). Comparative analysis of gene expression in forest and urban populations of *Anolis* lizards showed that rapid parallel regulatory adaptation to urban heat islands primarily resulted from selection for reduced and/or reversed heat‐induced plasticity, which is maladaptive in urban thermal conditions (Campbell‐Staton *et al*., 2021). A meta‐analysis of reciprocal transplant experiments indicated that adaptation to new environments only leads to genes losing their expression plasticity by genetic assimilation in rare cases (Chen & Zhang, 2024). In agreement, our results suggest that adaptation to temperature and daylength representative of their climate zone of origin maintained high expression plasticity of immunity genes in *A. thaliana* accessions. These insights will be valuable for assessing the adaptive potential of populations in the face of ongoing global climate change.

We identified two genes (*AT3G12910* and *AT5G64990*) the inactivation of which in the Col‐0 background leads to increased pathogen susceptibility following temperate acclimation but increased QDR following Mediterranean acclimation. Mutation in six other genotypes resulted in increased QDR following Mediterranean acclimation but no significant phenotype change following temperate acclimation. Conditionally beneficial or neutral mutations, that are deleterious in some environments but beneficial or neutral in others, have been reported in a wide range of organisms including plants (Elena & de Visser, 2003; Anderson *et al*., 2013). Recombinant inbred lines of the Brassicaceae plant *Boechera stricta* of diverse origin revealed that selection favored local alleles in contrasted environments, and 8.1% of the assessed markers showed evidence for conditional neutrality for the probability of flowering (Anderson *et al*., 2013). In the perennial grass *Panicum hallii*, an allele of the *FLOWERING LOCUS T‐like 9* locus from coastal ecotypes conferred a fitness advantage only in its local habitat but not at the inland site (Weng *et al*., 2022).

Loss‐of‐function alleles contribute to species adaptation (Olson, 1999; Xu & Guo, 2020) and have played an important role in crop domestication (Monroe *et al*., 2020). Naturally occurring loss‐of‐function variants are relatively rare, with an average 57 per genome in *A. thaliana* (Xu *et al*., 2019) and 18 per genome in soybean (Torkamaneh *et al*., 2021) but at the population level, they are found in 19% of soybean genes and 66% of *A. thaliana* genes. Conditionally neutral mutations are sufficient to drive patterns of local adaptation in simulations (Mee & Yeaman, 2019) and can emerge as a compensation to deleterious mutations (Farkas *et al*., 2022; Steinberg & Ostermeier, 2024). Simulations of long‐term evolution in changing environments produced complex gene regulatory networks with an increased rate of beneficial mutations, while a majority of mutations remain neutral (Crombach & Hogeweg, 2008). Patterns of local adaptation in *A. thaliana* (Fournier‐Level *et al*., 2011), the complexity of quantitative immunity networks (Delplace *et al*., 2020) and our focus on inoculation upregulated genes may explain the high proportion of conditionally beneficial loss‐of‐function we have identified. This finding suggests that targeted gene knockouts may be a promising strategy to improve climate resilience of plant immunity.

**Fig. 7:**
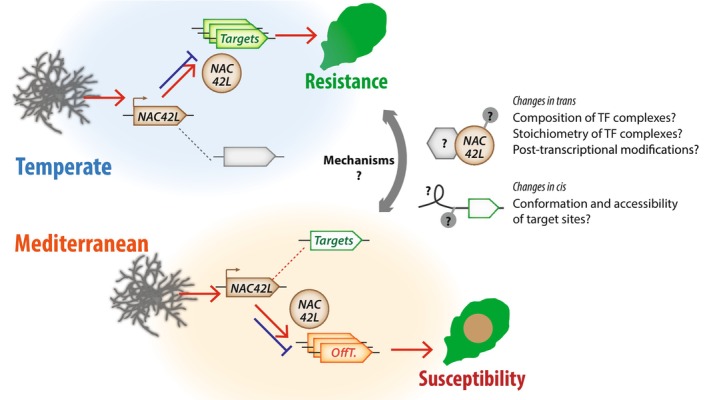
Schematic model of NAC42‐L activity following temperate and Mediterranean acclimation, including open perspectives on molecular mechanisms. Acclimation switches NAC42‐L from a positive regulator of quantitative disease resistance (temperate acclimation) to a negative regulator (Mediterranean acclimation). *Sclerotinia sclerotiorum* inoculation triggers the expression of *NAC42‐L* (brown arrow) and accumulation of NAC42‐L protein (brown circle), which regulates positively (red arrow) or negatively (blue blocked arrow) target genes. Upon temperate acclimation, NAC42‐L targets (green arrow) may positively contribute to quantitative disease resistance, while upon Mediterranean acclimation, NAC42‐L ‘off‐targets’ (orange arrows) mostly promote susceptibility to pathogens. The underlying molecular mechanisms are currently unknown and could potentially involve changes in *trans* and in *cis* (question marks on the right‐hand side of the scheme).

We identified *NAC42‐L* (*AT3G12910*) as an immunity factor following temperate acclimation but a susceptibility factor following Mediterranean acclimation. Its closest homolog, ANAC042/JUNGBRUNNEN1 (AT2G43000), was identified as a regulator of camalexin biosynthesis and positive regulator of resistance against the fungus *Alternaria brassicicola* (Saga *et al*., 2012), longevity (Wu *et al*., 2012) and tolerance to heat and drought (Shahnejat‐Bushehri *et al*., 2012; Ebrahimian‐Motlagh *et al*., 2017). In addition, exposure to 90 min at 37°C enhanced survival of *JUB1* overexpressors to a subsequent treatment at 45°C, compared with WT and *jub1–1* knock‐down seedlings (Shahnejat‐Bushehri *et al*., 2012). Molecular changes induced in plants by heat and other environmental signals persist longer than the signals themselves and modifies subsequent responses, phenomenon referred to as somatic environment memory (SEM). In this work, pathogen inoculations were done in standard conditions, indicating that some form of SEM of previous growth conditions had influenced plant immunity. The molecular mechanisms by which SEM mediates the priming of plant‐microbe interactions remain largely unknown. Our results implicated a switch in the *trans*criptional targets of NAC42‐L in this process, through mechanisms currently unknown (Fig. 7). Additional work will be needed to establish whether other transcription factors are similarly sensitive to acclimation and which of the identified NAC42‐L targets contribute to QDR against *S. sclerotiorum*. The underlying molecular bases may include variation in trans, through changes to the composition, stoichiometry and post‐transcriptional regulation of protein complexes including NAC42‐L, or variation in *cis* affecting the conformation and accessibility of target gene promoter regions (Fig. 7). Recent studies have identified chromatin state modifications as crucial components in the memory of repeated stress events in plants, particularly in response to heat, cold and drought priming (Crisp *et al*., 2016; Balazadeh, 2022; Liu *et al*., 2022). Environmental acclimation induces seasonal and diel changes in histone modifications like H3K27me3 (Nishio *et al*., 2020), which modulate both developmental plasticity and defense gene expression (Osborne, 2025). These epigenetic stress memories can create fitness trade‐offs, where priming for heat tolerance may reduce pathogen resistance (Liu *et al*., 2019b). Future investigations will aim at deciphering which molecular mechanisms mediate NAC42‐L target switch upon acclimation and what controls the duration and breadth of this switch.

Our results highlight rewiring of quantitative immunity gene networks as a key process in acclimation, with adverse consequence to QDR under warm Mediterranean‐like climates. We show that acclimation can reverse the contribution of genes upregulated by pathogen inoculation to the disease resistance phenotype. We identified several mutations mitigating the negative impact of Mediterranean acclimation on disease resistance, opening perspectives for the preservation of plant immunity functions in a warming climate context.

## Competing interests

None declared.

## Author contributions

AB and SR designed research; Natural accessions phenotyping: MD and JS; RNA‐seq sampling: JS and MD; RNA‐seq data analysis: JS, MD, FD and SR; Analysis of mutant lines genotype and phenotype: MD; NAC42‐L targets analyses: MD, PC‐S and MZ; Supervised and coordinated research: AB and SR; Wrote the paper: MD and SR.

## Disclaimer

The New Phytologist Foundation remains neutral with regard to jurisdictional claims in maps and in any institutional affiliations.

## Supporting information


**Dataset S1** Hierarchical gene network provided as a Cytoscape session file.

**Fig. S1** Climate data at sites in the distribution range of *Arabidopsis thaliana* and *Sclerotinia sclerotiorum* used for simulated climates in our experiments and at the site of Col‐0 accession origin.
**Fig. S2** Characterization of *Arabidopsis thaliana* natural accessions at the time of inoculation, following three acclimation regimes.
**Fig. S3** Plant responses to acclimation in the absence of *Sclerotinia sclerotiorum*.
**Fig. S4** Arabidopsis genes differentially expressed upon *Sclerotinia sclerotiorum* inoculation analyzed with relaxed thresholds.
**Fig. S5** Variations to differentially expressed genes upon inoculation according to acclimation regime.
**Fig. S6** Complementary information on the results of the analysis of variance to identify factors contributing the most to gene expression variation.
**Fig. S7** Properties of gene communities in a network of DEGs upon *Sclerotinia sclerotiorum* inoculation.
**Fig. S8** Visual aspect of 5‐wk‐old plants of mutant genotypes grown under Mediterranean and temperate acclimation.
**Fig. S9** Molecular characterization of *nac42‐like* mutant lines.
**Fig. S10** Preliminary characterization of selected NAC42‐L target genes.

**Table S1** List of oligonucleotide primers used in this work.
**Table S2** Raw data for *Arabidopsis thaliana* susceptibility phenotype in response to *Sclerotinia sclerotiorum* infection as a function of acclimation and genotype.
**Table S3** Normalized read counts for the 54 RNA‐seq samples and identification of expressed and differentially expressed genes.
**Table S4** Log_2_ fold change and *P*‐values for genes differentially expressed between acclimation regimes in non‐inoculated plants.
**Table S5** Gene ontologies enriched among genes differentially expressed between acclimation regimes in non‐inoculated plants.
**Table S6** Log_2_ fold change and *P*‐values for genes differentially expressed (inoculated samples vs mock‐treated samples as a reference).
**Table S7** Gene ontologies enriched in genes up‐ and downregulated in any accession following temperate, continental and Mediterranean acclimation.
**Table S8** Analysis of variance to determine the contribution of genotype, inoculation and acclimation to expression variance.
**Table S9** Expression values and differential expression of immunity‐related genes.
**Table S10** Content and representative properties of gene communities in the co‐expression network of *Arabidopsis thaliana* genes differentially expressed upon *Sclerotinia sclerotiorum* inoculation.
**Table S11** Gene ontologies enriched in major gene communities in the co‐expression network of *Arabidopsis thaliana* genes differentially expressed upon *Sclerotinia sclerotiorum* inoculation.
**Table S12** Raw data for *Arabidopsis thaliana* mutant lines susceptibility phenotype in response to *Sclerotinia sclerotiorum* inoculation.
**Table S13** Summary statistics for disease susceptibility of natural accessions and mutant lines inoculated by *Sclerotinia sclerotiorum* following temperate and Mediterranean acclimation.
**Table S14** Normalized read counts for the Col‐0, *nac42‐L1* and *nac42‐L2* inoculated samples used for the identification of NAC42‐L putative target genes.
**Table S15** Log_2_ fold change and *P*‐values for genes differentially expressed in *nac42‐L1* and *nac42‐L2* plants relative to Col‐0.
**Table S16** Gene ontologies enriched in NAC42‐L putative target genes identified by RNA‐seq of *nac42‐L* mutants.
**Table S17** List of target sequences and genes harboring the DAP‐seq motif bound by AT3G12910 identified by FIMO.
**Table S18** Normalized read counts and hierarchical cluster assignment for the 210 DEGs in both *nac42‐L* mutants with NAC42‐L binding motifs in their promoter.
Please note: Wiley is not responsible for the content or functionality of any Supporting Information supplied by the authors. Any queries (other than missing material) should be directed to the *New Phytologist* Central Office.

## Data Availability

Raw RNA‐seq reads data for natural accessions and *nac42‐L* mutants generated in this work are available from the European Nucleotide Archive (ENA) with accession no. PRJEB88178. Processed gene expression files are available at doi: 10.6084/m9.figshare.29350589.
