# Supplementary material for: Arabidopsis acclimation to daily environmental fluctuations converts a defense response regulator into a susceptibility factor toward Sclerotinia
Source: New Phytol. 2026 Mar 7;250(4):2516–34. doi: 10.1111/nph.71053 (PMC13103438; doi:10.1111/nph.71053)
Supplement: Supplementary file 2 — Fig. S1 Climate data at sites in the distribution range of Arabidopsis thaliana and Sclerotinia sclerotiorum used for simulated climates in our experiments and at the site of Col‐0 accession origin. Fig. S2 Characterization of Arabidopsis thaliana natural accessions at the time of inoculation, following three acclimation regimes. Fig. S3 Plant responses to acclimation in the absence of Sclerotinia sclerotiorum. Fig. S4 Arabidopsis genes differentially expressed upon Sclerotinia sclerotiorum inoculation analyzed with relaxed thresholds. Fig. S5 Variations to differentially expressed genes upon inoculation according to acclimation regime. Fig. S6 Complementary information on the results of the analysis of variance to identify factors contributing the most to gene expression variation. Fig. S7 Properties of gene communities in a network of DEGs upon Sclerotinia sclerotiorum inoculation. Fig. S8 Visual aspect of 5‐wk‐old plants of mutant genotypes grown under Mediterranean and temperate acclimation. Fig. S9 Molecular characterization of nac42‐like mutant lines. Fig. S10 Preliminary characterization of selected NAC42‐L target genes. [file NPH-250-2516-s001.pdf]

New Phytologist Supporting Information

Article title: ***Arabidopsis* acclimation to daily environmental fluctuations converts a defense response regulator into susceptibility factor towards *Sclerotinia***

Authors: Marie Didelon, Justine Sucher, Florent Delplace, Pedro Carvalho-Silva, Matilda Zaffuto, Adelin Barbacci, Sylvain Raffaele

Article acceptance date: 7 February 2026

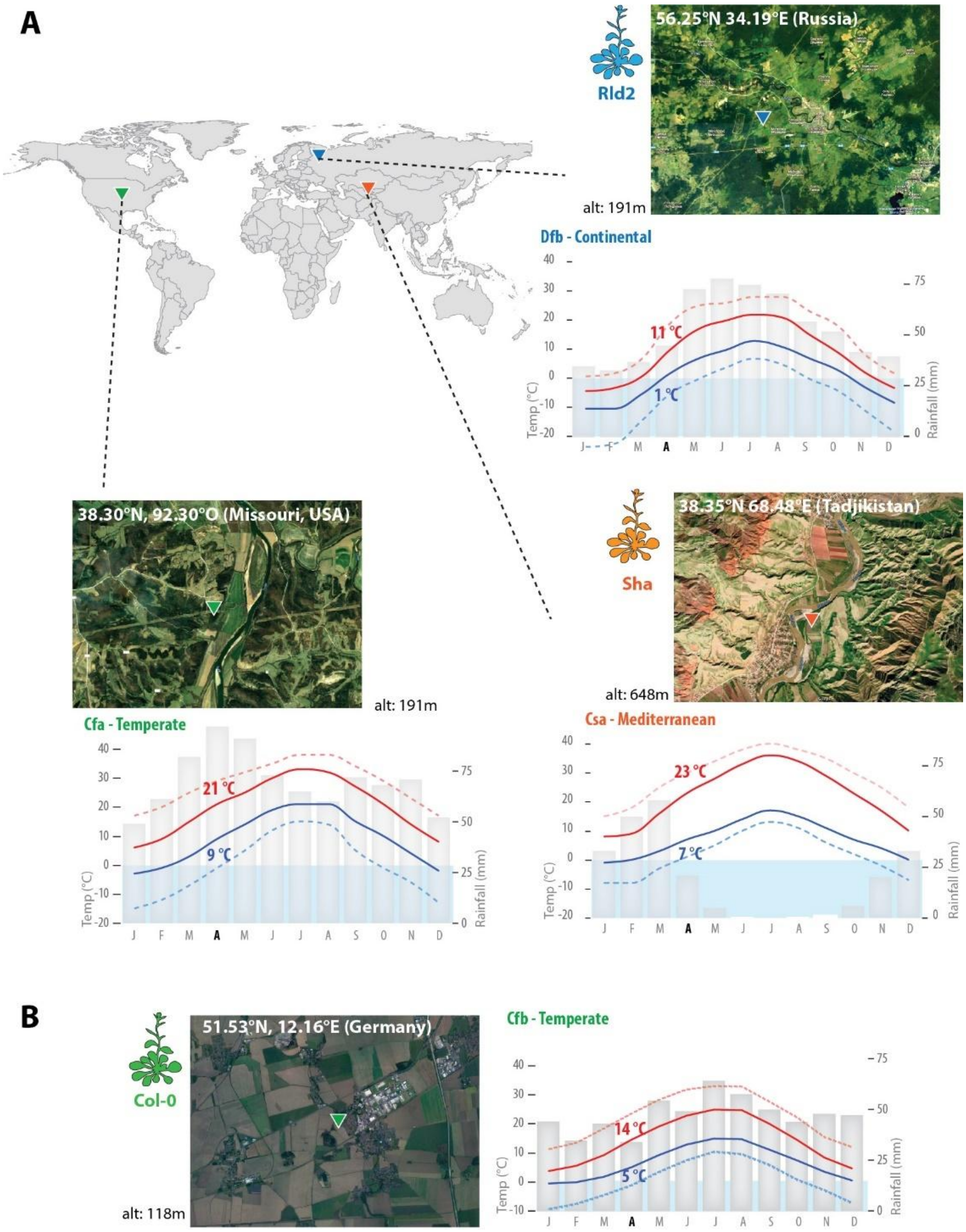

Supplementary Figure S1. Climate data at sites in the distribution range of *A. thaliana* and *S. sclerotiorum* used for simulated climates in our experiments (A) and at the site of Col-0 accession origin (B). Data come from an ERA5T model of 30-year average of hourly simulations collected from meteoblue.com. Satellite views of the designated coordinates were obtained from Google Maps. Grey bars show monthly precipitations in mm, red lines are mean daily maximum (plain) and hot days maximum (dotted), blue lines are mean daily minimum (plain) and cold nights minimum (dotted). Values for April are labelled. The corresponding Köppen-Geiger climate was obtained from climate-data.org and coded as follows: Csa, hot summer Mediterranean climate; Cfa, humid subtropical climate; Cfb, Temperate oceanic climate or subtropical highland climate; Dfa, Hot-summer humid continental climate. Alt., altitude; Temp., Temperature.

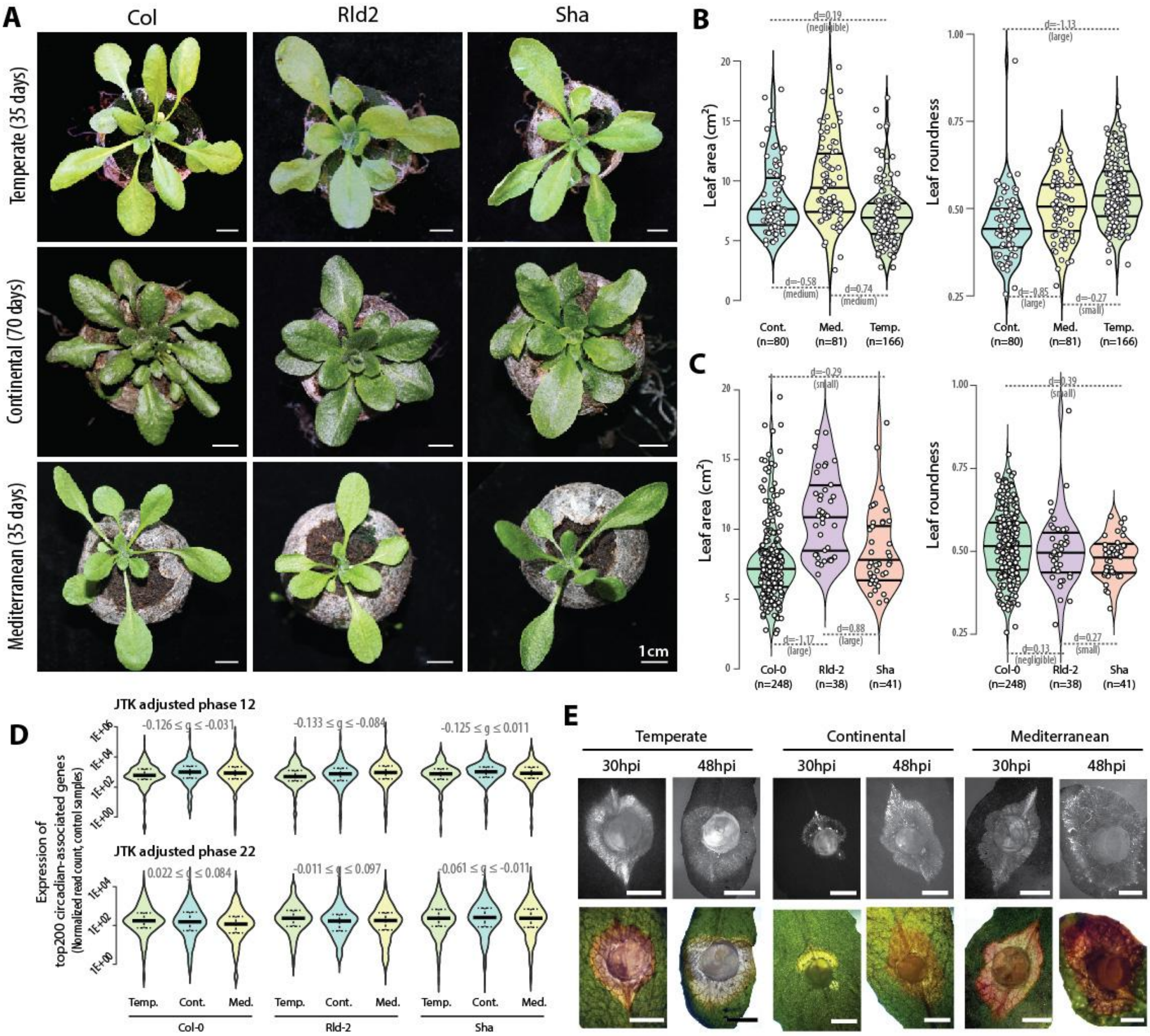

Supplementary Figure S2. Characterization of *A. thaliana* natural accessions at the time of inoculation, following three acclimation regimes. (A) representative pictures of plants at the time of inoculation. Bar shows 1 cm. (B, C) Measures of leaf area (in cm²) and leaf roundness for natural accessions according to acclimation regimes (B) and genotype (C). Effect size were estimated using Cohen's d. Horizontal bars in violin plots indicate 1st quartile, median and 3rd quartile. Measurements were collected in 4 to 6 independent experiments per acclimation regime. (D) Expression of top200 circadian-associated genes identified by Reynolds *et al.*, 2025 phased at 12 and 22 hours, across genotypes and acclimation conditions. Values are median normalized read count. Plain lines show median values, dotted lines show first-third quantile range. Acclimation effect size is indicated for each genotype as Hedge's g value intervals. (E) Representative pictures of Col-0 plants inoculated by *S. sclerotiorum* 1980 expressing GFP. Plants were acclimated under temperate, continental and Mediterranean regimes and imaged at 30 and 48 hours post inoculation. Fluorescence pictures (top) and bright field images (bottom) are provided. Bar=5 mm.

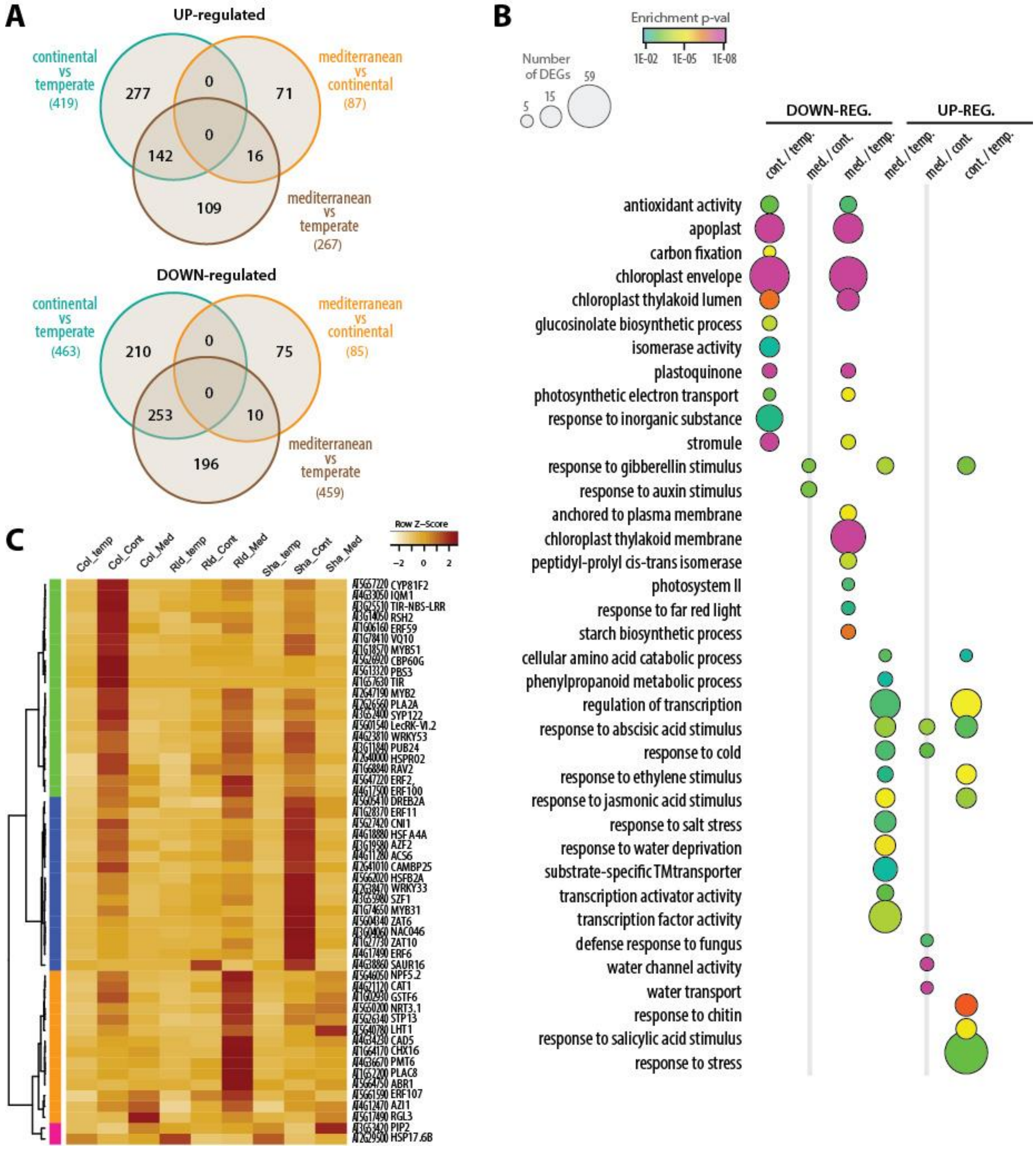

Supplementary Figure S3. Plant responses to acclimation in the absence of *S. sclerotiorum*. (A) Distribution of differentially expressed genes (DEGs) in non-inoculated plants in pairwise acclimation comparisons. Numbers between brackets indicate the total number of DEGs per comparison. (B) Gene ontologies enriched among down-regulated (left) and up-regulated (right) DEGs in pairwise acclimation comparisons with non-inoculated plants. Circles are sized according to the number of DEGs per GO and colored according to the enrichment p-value (Hypergeometric test with Benjamini-Hochberg correction). (C) Relative expression of 52 immunity-related genes differentially expressed in comparisons between non-inoculated acclimated plants. Cont., continental; med., Mediterranean; temp., Temperate.

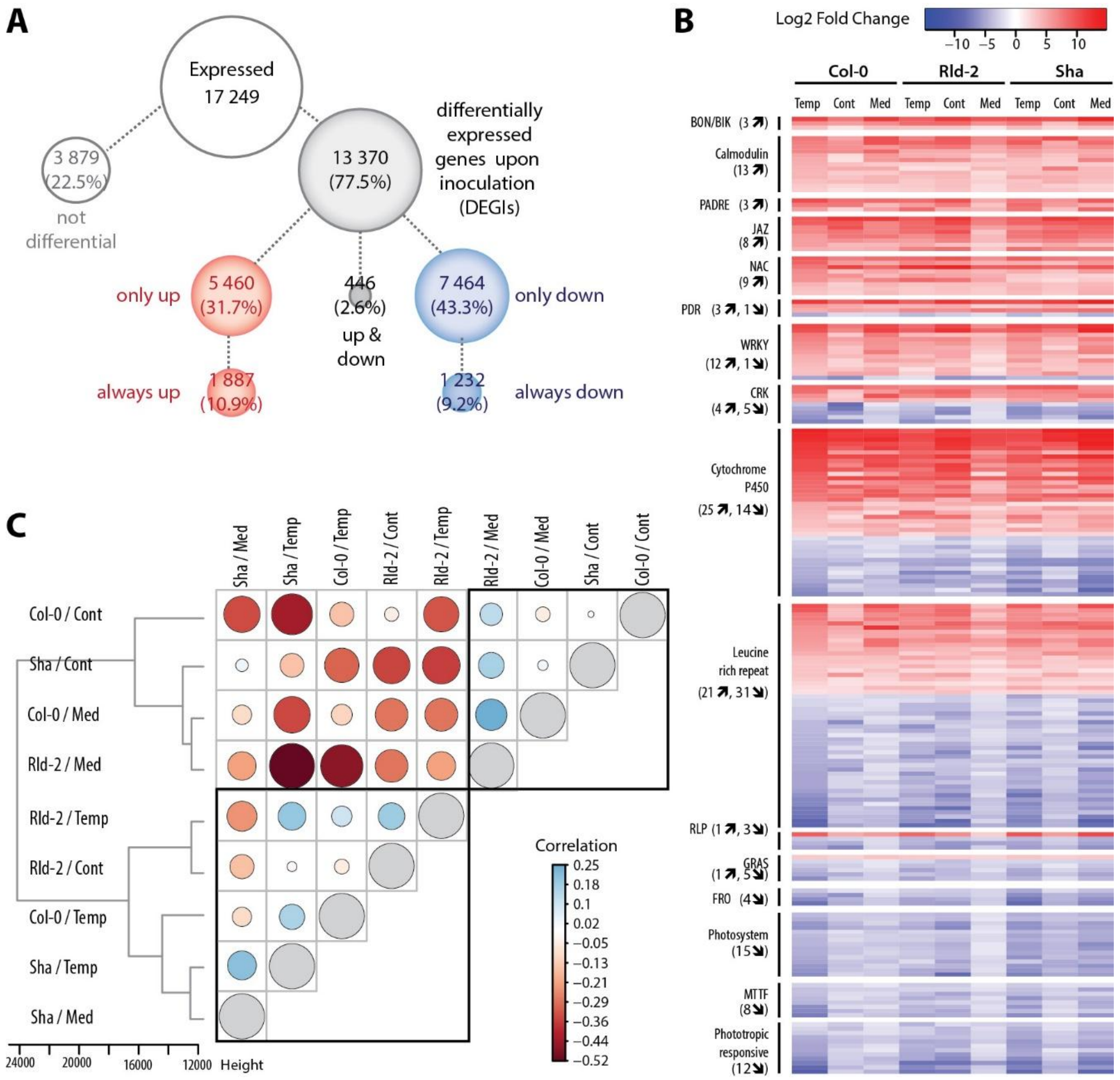

Supplementary Figure S4. ***Arabidopsis*** **genes differentially expressed upon** ***S. sclerotiorum*** **inoculation analyzed with relaxed thresholds.** Analysis of genes differentially expressed upon inoculation at |Log2 Fold Change|≥1.5, adjusted p-val<0.1, using non-inoculated plants as reference in each of nine conditions (three climate priming, times three plant genotypes). **(A)** Identification of 13 370 differentially expressed genes (DEGs) upon inoculation. In conditions where they are differential, 5 460 DEGs were upregulated only, 7 464 were down-regulated only, and 446 were either up or down-regulated. We detected 1 887 DEGs upregulated in all nine conditions and 1 232 DEGs down-regulated in all nine conditions, representing 10.9% and 9.2% of the expressed genes respectively. These 3 119 genes are differentially expressed in a consistent manner regardless of plant genotype and acclimation, they can therefore be regarded as a core transcriptome responsive to *S. sclerotiorum* in *A. thaliana*. **(B)** Heatmap of Log2 fold change for 200 genes forming major functional groups including DEGs always up and always down (numbers indicated between brackets). Major functional groups in the core transcriptome included the PRR-associated *BOTRYTIS-INDUCED KINASE 1* (*BIK1*), *BONZAI1-ASSOCIATED PROTEIN (BAP) 1* and *2*, members of the Calmodulin (CaM) and CaM-binding, the pathogen and abiotic stress response, cadmium tolerance, disordered region-containing (PADRE), the jasmonate-zim-domain proteins (JAZ), and the NAC-domain transcription factor families, with all core DEGs being upregulated by *S. sclerotiorum* inoculation. Ferric reduction oxidases (FRO), Mitochondrial transcription termination factors (MTTF), Photosystems I and II, phototropin and

phototropic-responsive NPH3 genes showed all core DEGs downregulated. The pleiotropic drug resistance (PDR), cysteine-rich receptor-like protein kinase (CRK), WRKY and GRAS transcription factor, cytochrome P450, Leucine-rich repeat (LRR), receptor like protein (RLP) families included several core genes responsive to *S. sclerotiorum* either consistently up- or down-regulated. **(C)** Distance tree and correlation matrix showing the similarity in the 13 370 DEGs regulation across conditions. Tree based on Manhattan distance between samples and Ward clustering, correlation values shown by bubbles are Spearman rank correlations calculated using LFC for 13 370 DEGs in each condition. Transcriptomes measured after priming under temperate acclimation clustered together and with the transcriptome of Rld-2 primed under continental acclimation and that of Sha under Mediterranean acclimation. The remaining four conditions formed a second cluster. There was no clear clustering based on genotype or climates alone, suggesting a significant interaction between these factors. Cont, continental; Temp, temperate; Med, Mediterranean.

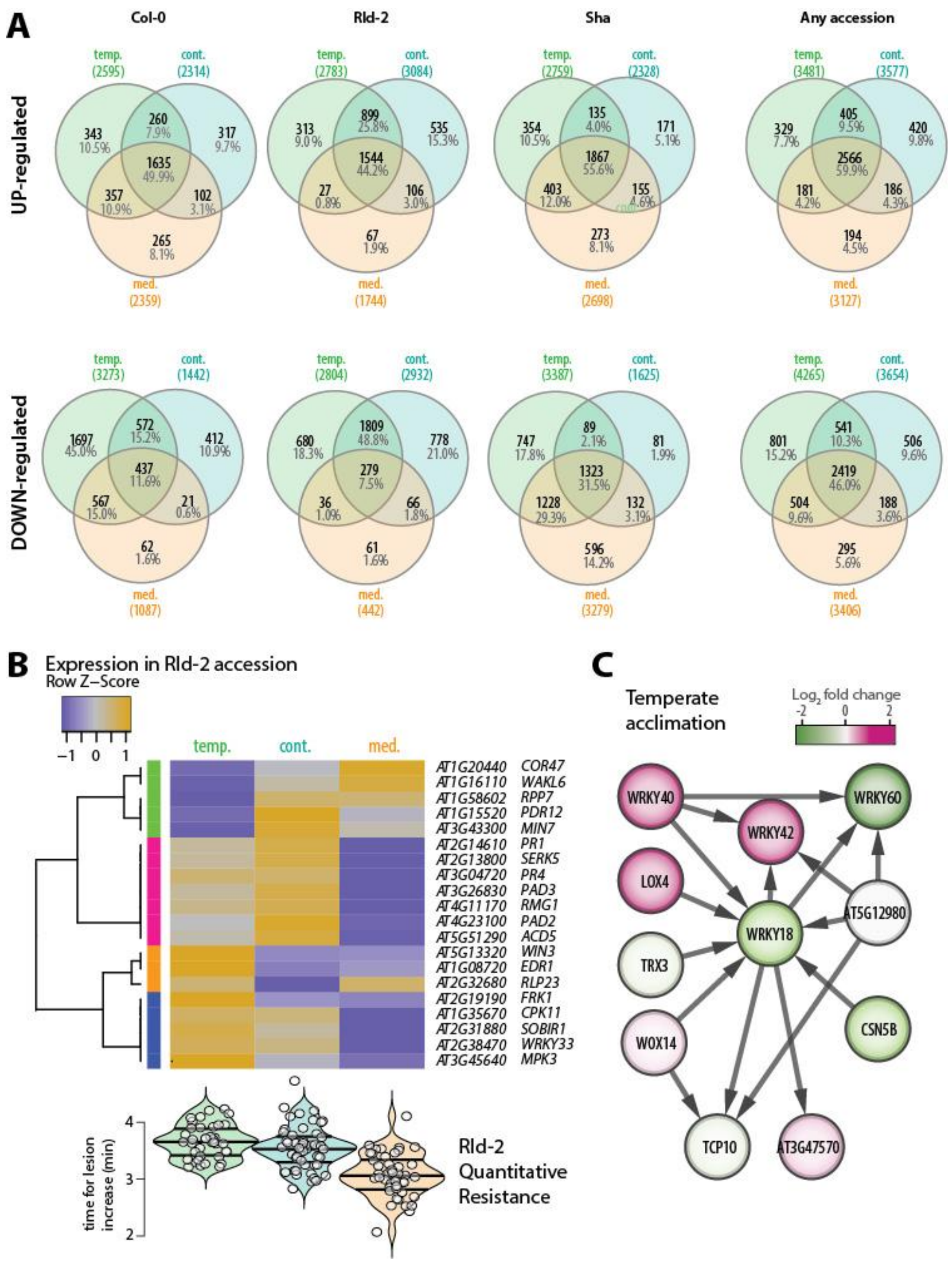

Supplementary Figure S5. Variations to differentially expressed genes (DEGs) upon inoculation according to acclimation regime. (A) Genes were considered significantly differential for |Log2 Fold Change|≥2 and Bonferroni-adjusted p-val<1E-4. The number of DEGs is shown in black, the corresponding % of DEGs per accession is shown in grey, the total number of DEGs per acclimation regime is indicated between brackets. **(B)** Variation in LFC upon inoculation for 20 genes associated with plant immunity in the Rld-2 accession according to acclimation regime. The bottom violin plots recall the quantitative disease resistance phenotype for the corresponding plants. The middle line shows median value, upper and lower lines show 1st and 3rd quartile values respectively. Rld-2 is shown as an illustration for showing the most dramatic phenotypic alterations upon Mediterranean acclimation. Cont., continental; med., Mediterranean; temp., temperate. **(C)** Subset of the gene and protein network connected to the transcription factor WRKY18 through co-expression and protein-protein interaction relationships. Circles representing genes are colored according to their expression (Log2 fold change upon pathogen inoculation) after temperate acclimation.

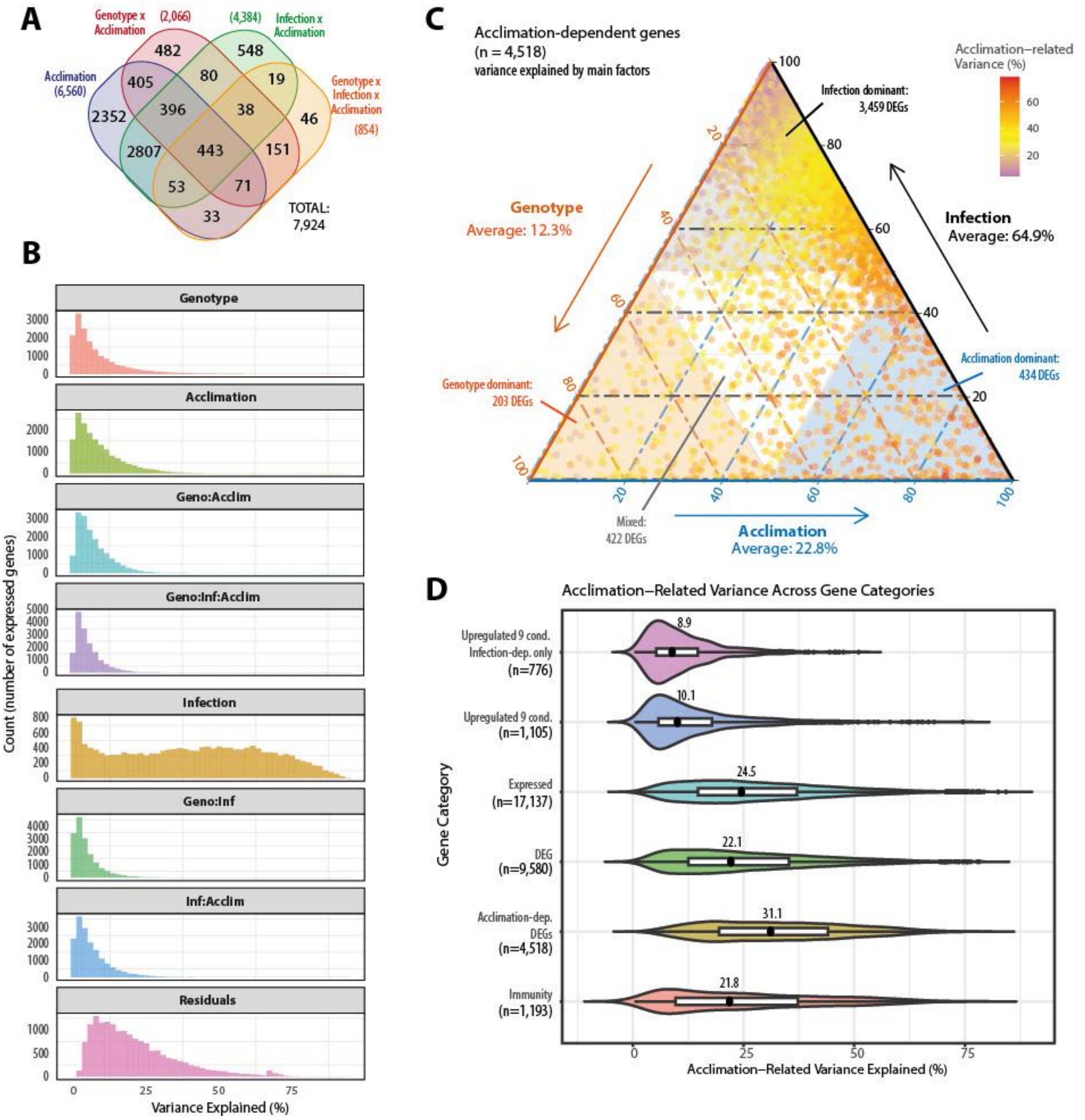

Supplementary Figure S6. Complementary information on the results of the analysis of variance (ANOVA) to identify factors contributing the most to gene expression variation. (A) Distribution of acclimation-dependent genes identified by ANOVA according to the factors explaining gene expression variance. Number correspond to genes identified among all 17,137 expressed genes. (B) Distribution of expressed genes according to the variance explained by each factor and interactions between factors. (D) Relative importance of the three tested factors (Genotype, infection, acclimation) in gene expression variance for differentially expressed genes (DEGs) with a significant dependence to acclimation (adjusted p-val<2E-03). Data points are colored according to the sum of acclimation-related variance (acclimation, genotype x acclimation, infection x acclimation and genotype x infection x acclimation interactions). Genes closer to the acclimation vertex have higher acclimation main effect, but color shows total acclimation involvement including interactions. Dominant factors are factors responsible for at least 50% of main effects variance. (D) Distribution of acclimation-related variance across gene categories. Boxplots show median, first and third quartile values, median values are labelled. Cond., conditions; dep. Dependent.

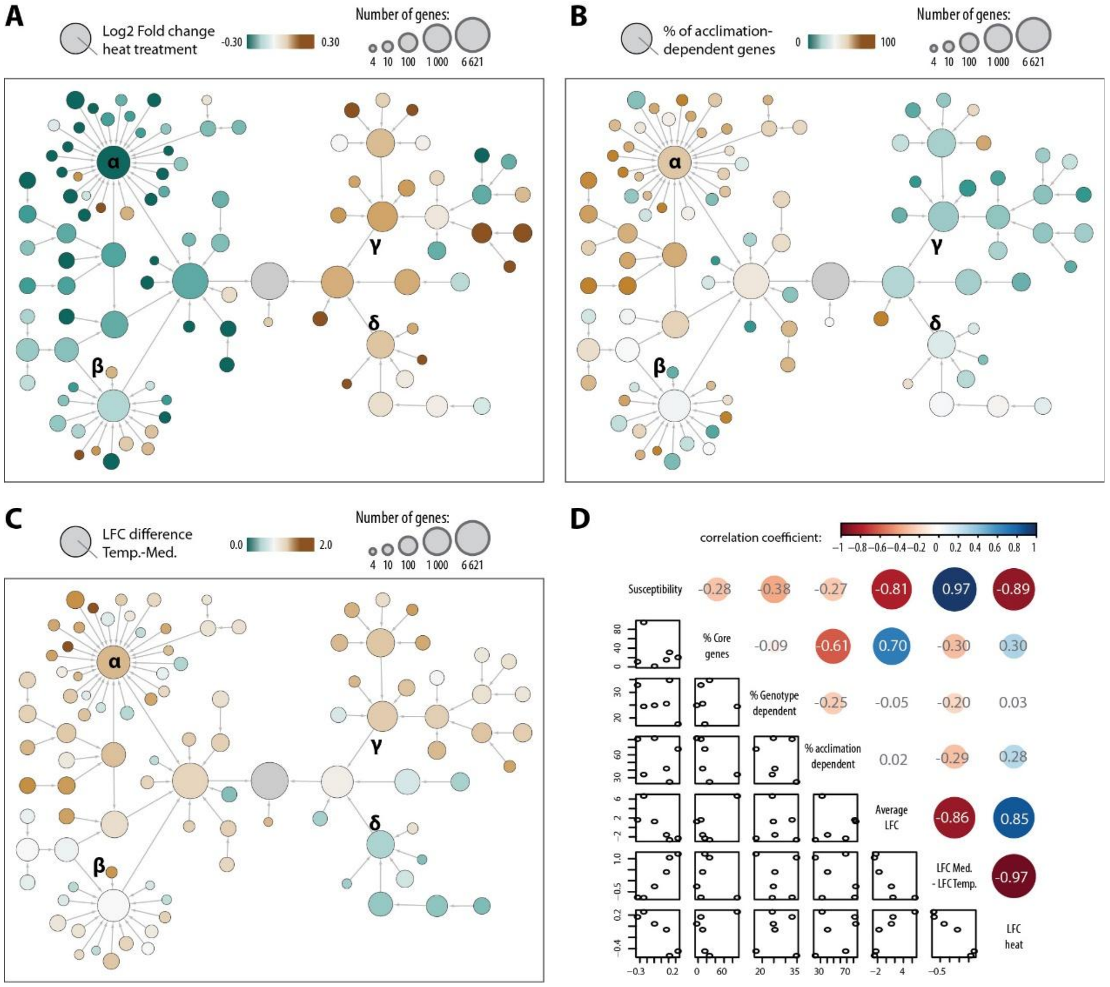

Supplementary Figure S7. Properties of gene communities in a network of DEGs upon S. sclerotiorum inoculation. (A) We mapped the average gene LFC upon heat treatment reported in a recent meta-analysis (Guo *et al*. 2021). Genes from communities γ and δ showed a trend for up-regulation upon heat treatment (average LFC 0.26 and 0.17 respectively) while genes from communities α and β were rather down-regulated (average LFC -0.52 and -0.45 respectively). **(B)** We mapped the percentage of gene from each community considered acclimation dependent based on our ANOVA analysis. Communities γ and δ had a low proportion of acclimation-dependent genes while community α had a majority of acclimation-dependent genes. **(C)** Considering the clear phenotypic effect of Mediterranean acclimation, which had the highest day temperature and highest daily thermal amplitude, we mapped the average LFC variation between plants acclimated under temperate and Mediterranean climates. Average LFC variation was >1.0 for communities α and γ but <-0.7 for community δ. (D) To summarize these analyses, we calculated the correlation between properties of the six largest gene communities. We observed a clear correlation between association with susceptibility phenotype and LFC variation upon temperate and Mediterranean acclimation (0.97), and anti-correlation with average LFC upon *S. sclerotiorum* inoculation (-0.81 and -0.86). This suggested that in our experiments, differential gene expression mostly associated with a decrease in plant susceptibility which is strongly altered upon Mediterranean acclimation.

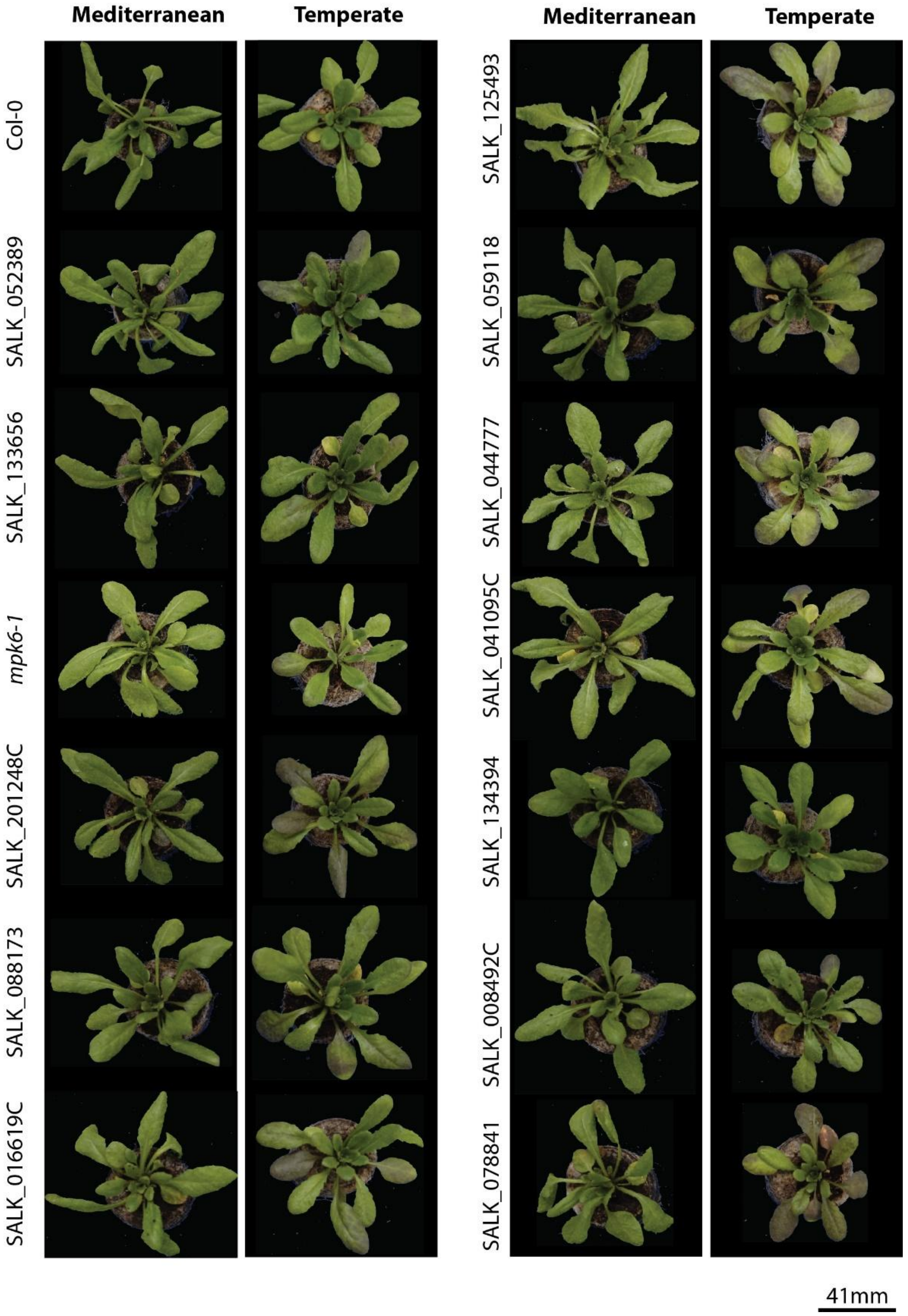

Supplementary Figure S8. Visual aspect of 5-week old plants of mutant genotypes grown under Mediterranean and temperate acclimation.

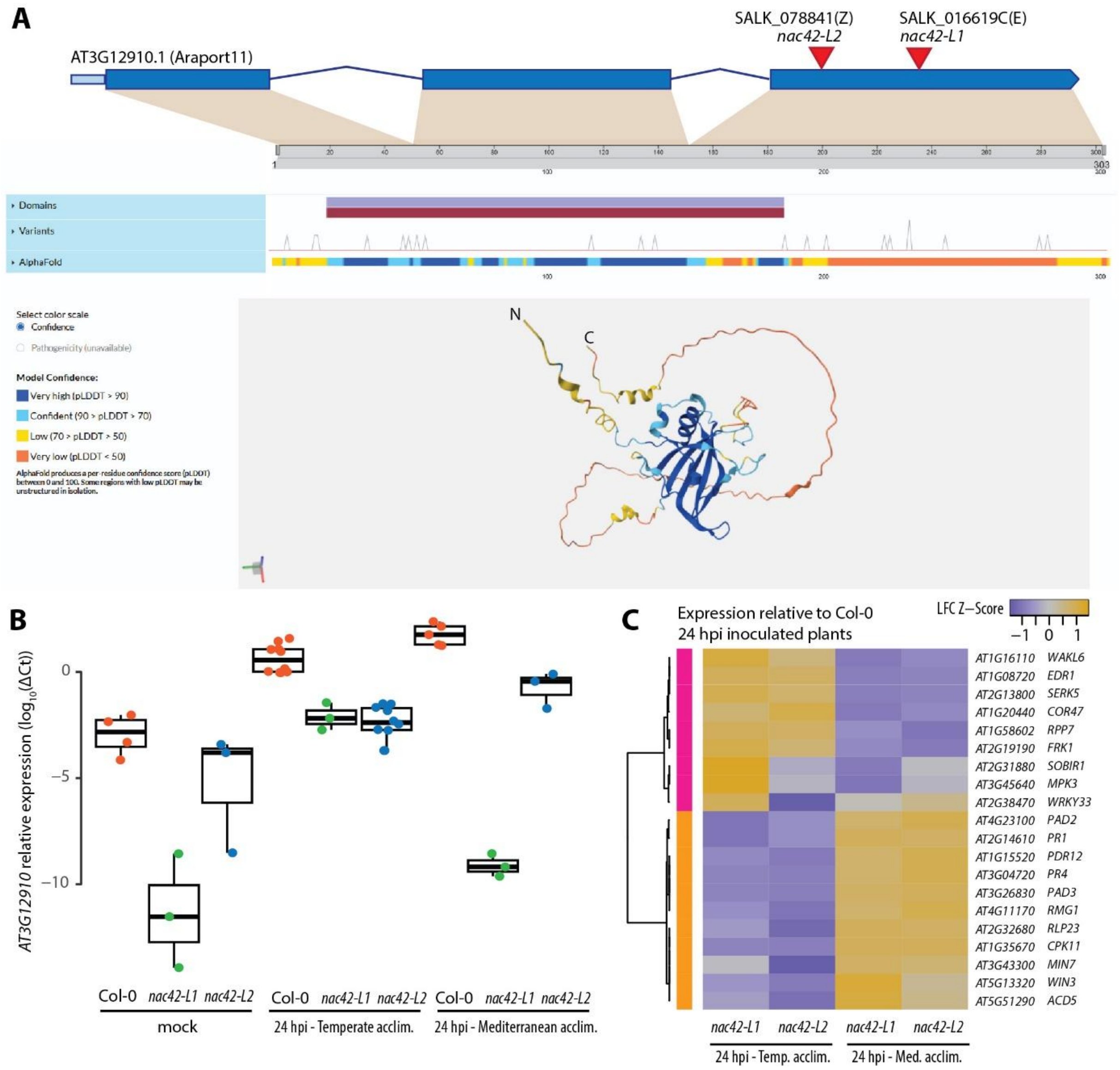

Supplementary Figure S9. Molecular characterization of *nac42-like* mutant lines. (A) map of *NAC42-like* gene structure with exons shown as plain boxes, introns as broken connectors, untranslated regions as empty boxes. The position of transfer-DNA insertions, confirmed as homozygous by PCR and amplicon sequencing are shown as red triangles. Correspondence with the protein domains and regions are shown as brown connectors. The predicted NAC-L protein structure by AlphaFold 2.0 is shown colored by confidence (pLDDT) score. The two T-DNA insertions reside in the C-terminal exon 3 which encodes the C-terminus of the NAC domain and a ~100 amino-acids long disordered region (yellow and orange regions). **(B)** Expression of *NAC42-L* in Col and *nac42-like* mutant plants measured by quantitative RT-PCR. Values shown are $\log_{10}\Delta Ct$ relative to reference gene *AT2G28390* in 3-8 independent biological replicates. **(C)** Variation in LFC between *nac42-like* mutants and Col-0 for 20 genes associated with plant immunity according to acclimation regime. Acclim., acclimation; med., Mediterranean; temp., temperate.

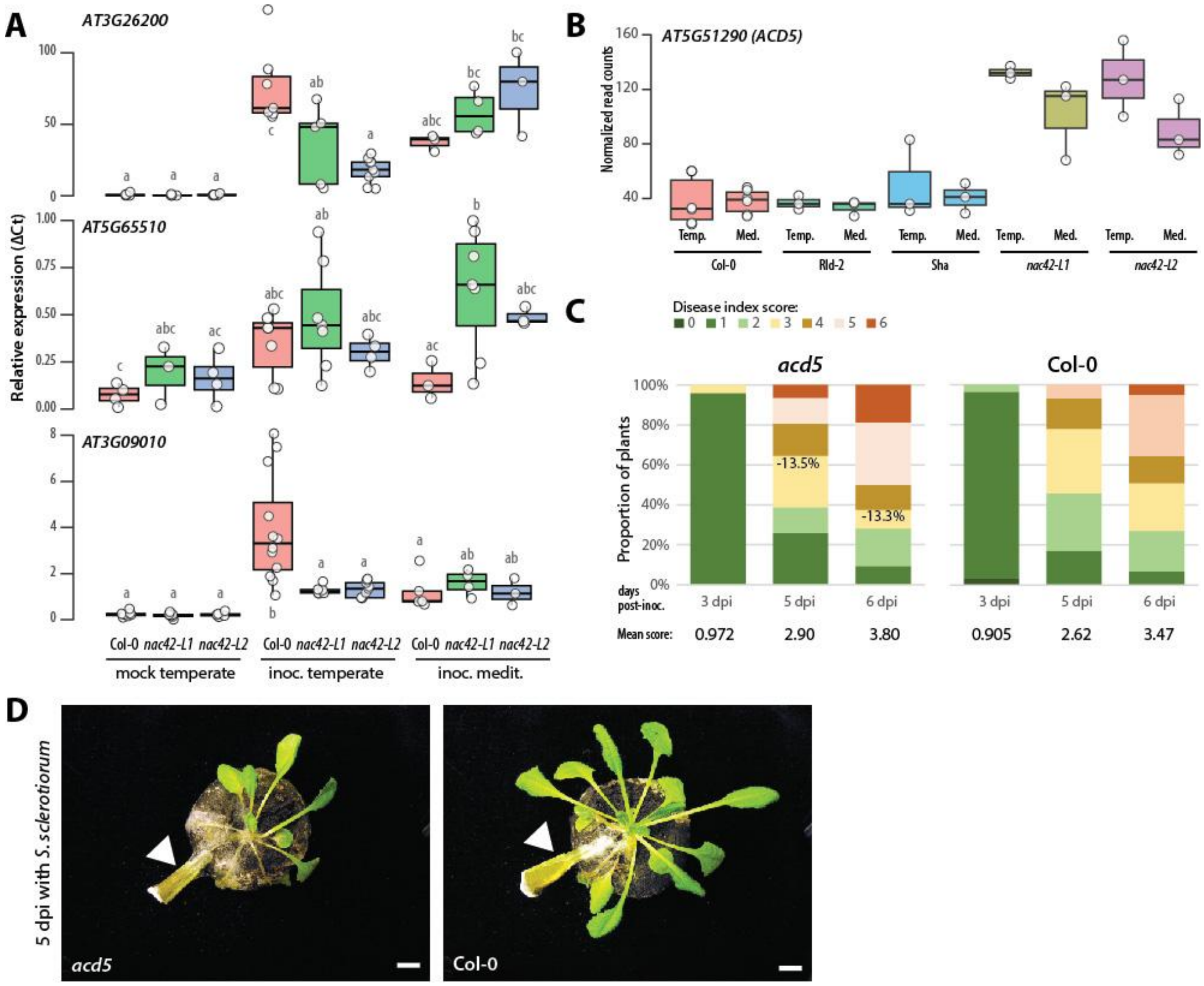

Supplementary Figure S10. Preliminary characterization of selected NAC42-L target genes. (A) Quantitative reverse-transcription PCR (Q-RT-PCR) analysis of the expression of three NAC42-L target genes. The expression of these genes obtained by RNA-seq is provided in Fig 6E. Values shown are from 3 to 12 independent biological replicates measured in 2 or 3 technical replicates each. Letters indicate significance groups determined by Tukey HSD test. Inoc., inoculated. **(B)** Expression in read count per million of the NAC42-L target gene *AT5G51290* (*ACD5*) in inoculated samples. Boxplots show median (plain line), first and third quartile (box) and 1.5 the interquartile range (whiskers). Letters indicate Tukey HSD significance groups. Temp., temperate; Med., Mediterranean. **(C)** Preliminary characterization of the disease resistance phenotype of an *acd5* mutant line, using disease score index. The homozygous *acd5* mutant line in the Col-0 background was kindly provided by Prof. Jean T. Greenberg (Uni. Of Chicago, USA) (Liang *et al*. 2003). Symptoms were scored on 29 to 51 plants per genotype. Labels on the *acd5* histograms indicate the % difference compared to wild type for plants with score below or equal to 3. Dpi, days post-inoculation. **(D)** representative pictures of plants 5 days post inoculation with *S. sclerotiorum*. The inoculated leaf is indicated by a triangle. Bar = 1 cm.

SUPPLEMENTARY REFERENCES

Guo, M., Liu, X., Wang, J., Jiang, Y., Yu, J., & Gao, J. (2021). Transcriptome profiling revealed heat stress-responsive genes in *Arabidopsis* through integrated bioinformatics analysis. *Journal of Plant Interactions*, *17*(1), 85–95. https://doi.org/10.1080/17429145.2021.2014580

Liang H, Yao N, Song JT, Luo S, Lu H, Greenberg JT. Ceramides modulate programmed cell death in plants. Genes Dev. 2003 Nov 1;17(21):2636-41. doi: 10.1101/gad.1140503. Epub 2003 Oct 16. PMID: 14563678; PMCID: PMC280613.

Reynolds, C., Colmer, J., Rees, H. *et al.* Machine learning models highlight environmental and genetic factors associated with the *Arabidopsis* circadian clock. *Nat Commun* **16**, 7223 (2025). https://doi.org/10.1038/s41467-025-62196-w
